# A family of auxin conjugate hydrolases from *Solanum lycopersicum* and analysis of their roles in flower pedicel abscission

**DOI:** 10.1186/s12870-019-1840-9

**Published:** 2019-06-03

**Authors:** Xin Fu, Zihang Shi, Yun Jiang, Lingling Jiang, Mingfang Qi, Tao Xu, Tianlai Li

**Affiliations:** 1 Horticulture Department, Shenyang Agricultural University, No. 120 Dongling Road, Shenhe District, Shenyang, 110866 Liaoning China; 2 Key Laboratory of Protected Horticulture of Ministry of Education, No.120 Dongling Road, Shenhe District, Shenyang, 110866 Liaoning China; 3 Shenyang Entry-exit Inspection and Quarantine Bureau, No.433 Danan street, Shenhe District, Shenyang, 110016 Liaoning China

**Keywords:** Auxin conjugate hydrolases, Indole-3-acetic acid, *Solanum lycopersicum*, Flower pedicel abscission

## Abstract

**Background:**

Auxin conjugates are hydrolyzed to release free auxin to ensure defined cellular auxin levels or gradients within tissues for proper development or response to environmental signals. The auxin concentration in the abscission zone (AZ) is thought to play an important role in mediating the abscission lag phase.

**Results:**

In this study, the full cDNA sequences of seven tomato ILR1-like *SlILL* genes were identified and characterized, All SlILLs were found to have auxin conjugate hydrolysis activity. The effects of different auxin conjugates on abscission identified IAA-Ile as a candidate to determine the auxin conjugate and auxin conjugate hydrolysis functions in abscission. Treatment of pedicel explants with IAA-Ile for different times showed that application before 6 h could effectively delay abscission. IAA-Ile pre-incubation for 2 h was sufficient to inhibit abscission. These results showed that there is not sufficient auxin conjugates in the AZ to inhibit abscission, and the optimal time to inhibit abscission by the application of exogenous auxin conjugates is before 6 h. Treatment with cycloheximide (CHX, a protein biosynthesis inhibitor) indicated that de novo synthesis of auxin conjugate hydrolases is also required to delay abscission. During abscission, *SlILL1*, *5*, and *6* showed abscission-related gene expression patterns, and *SlILL1*, *3*, *5*, *6*, and *7* showed increasing expression trends, which collectively might contribute to delay abscission. Silencing the expression of *SlILL1*, *3, 5*, *6*, and *7* using virus-induced gene silencing showed that SlILL1, 5, and 6 are major mediators of abscission in tomato.

**Conclusions:**

In the process of abscission, auxin inhibition is concentration dependent, and the concentration of auxin in the AZ was regulated by hydrolyzed auxin conjugates. SlILR1, 5, and 6 play a key role in flower pedicel abscission.

**Electronic supplementary material:**

The online version of this article (10.1186/s12870-019-1840-9) contains supplementary material, which is available to authorized users.

## Background

Indole-3-acetic acid (IAA) is an essential regulator of many aspects of plant growth and development [1]. In most tissues, auxin responses are concentration dependent, and different tissues respond in a distinct manner to varying amounts of exogenous auxins [2, 3]. Low IAA concentrations stimulate growth, while high concentrations can be inhibitory to organ development or are toxic to the plant [4, 5]. In plant organs, local auxin concentrations are altered to maintain optimum concentrations using various mechanisms, such as biosynthesis, degradation, transport, and conjugate formation. Auxin conjugates are formed to turn free auxin into bound forms, in which the carboxyl group is conjugated to sugars via ester linkages, or to amino acids or peptides via amide linkages [6, 7]. However, little is known about the spectrum of auxin conjugates present in a given tissue because of the difficulty in analyzing individual conjugates. The function of auxin conjugates has been mainly determined using mutant analysis, implying that auxin conjugates are connected with development of the embryo, shoot, and vasculature through their possible roles in storage and transport of auxins and protecting free IAA from degradation [2, 8]. The content of free IAA and that stored as conjugates are maintained in a balance. Under normal conditions, only very small amounts, approximately 5%, of the auxin in a plant is free; the majority of the auxin is conjugated into amide-linked and ester-linked conjugates of IAA as storage forms [5, 9, 10]. However, only a fraction of the IAA conjugates, such as IAA-Ala, IAA-Leu, IAA-Phe, and IAA-Ile are hydrolyzed back to free IAA via auxin amino acid conjugate hydrolyses, and most intriguingly, IAA-Ala can act as an auxin without being hydrolyzed to release free IAA in tomato cell culture, whereas the amino acid conjugates IAA-Asp and IAA-Glu are thought to be precursors for a degradation pathway. Thus the different IAA conjugates vary in their effects on plant development and responses, such as seed germination and root elongation [2, 11–13].

The hydrolysis of amino acid-type IAA conjugates has been studied in great detail, revealing gene families from various plant species with distinct, yet overlapping, substrate specificities for various conjugates of both IAA and indole-3-butyric acid (IBA) [4, 12, 14, 15]. In *Arabidopsis,* ILR1 cleaves IAA-Leu and IAA-Phe preferentially, whereas the ILL1, ILL2, and resistant IAR3 enzymes prefer IAA-Ala as a substrate. ILR1, IAR3, ILL1, and ILL2 encode active IAA-amino acid hydrolases, and there are three additional amidohydrolase-like genes (*ILL3*, *ILL5*, and *ILL6*). Single mutants of *ILL1* and *IAR3* show a normal plant phenotype. MtIAR31, − 32, − 33, and − 34 from *Medicago truncatula* have hydrolytic activity against IAA-aspartate and IBA-alanine. Only MtIAR36 is highly active against IAA-glycine, −alanine, and -isoleucine (IPA) [4, 14, 16, 17]. The different enzyme activities and expression patterns make it reasonable to deduce that induction of one specific hydrolytic activity in response to a particular challenge, suggesting that various conjugate hydrolases might supply free IAA in response to a variety of needs [1, 2, 7].

When the abscission zone (AZ) auxin concentration drops below a certain threshold, abscission is initiated [18–20]. The AZ auxin is mostly derived from de novo synthesis in the young organ. Removal of flowers or leaves to deplete the AZ auxin source will induce abscission rapidly compared with the normal abscission caused by the absence of pollination or pathogen attack [5, 7, 21]. However, even in this process, abscission is not initiated immediately, and the delay stage can last for at least two hours [20, 22]. This is a problem if stored auxin conjugates in the AZ are not converted to active free IAA, because free IAA is very unstable and would oxidase or degrade in several minutes. Previous studies have shown that there are peaks of increased IAA levels after flower removal during abscission, which further confirms the view that hydrolysis of auxin conjugates plays an important role in mediating organ abscission [20, 22]. However, because the ILL protein family members in tomato are not well characterized, little information is available concerning the function of auxin conjugate hydrolysis in mediating flower pedicel abscission. The auxin conjugate hydrolase family members are present in both monocot and dicot plants [22]. The entire gene families have been well characterized in some species; for example, seven members of this family have been identified from Arabidopsis, six from *Brassica rapa* [15], and six from *Medicago truncatula* [14].

In this study, we isolated the full cDNA sequences of seven novel *SlILL*s from tomato (*Solanum lycopersicum* L.). Enzyme assays confirmed that these SlILLs have auxin conjugate hydrolysis activity. Treatment with IAA-Ile for various times in the presence of cycloheximide (CHX, a protein biosynthesis inhibitor) showed that there is inadequate auxin conjugate and auxin conjugate hydrolyzing enzyme levels in the AZ to inhibit abscission, and the optimal time to inhibit abscission by applying exogenous auxin conjugate is before 6 h. Virus-induced gene silencing (VIGS) of *SlILL1*, *3*, *5*, *6* and *7* expression showed that the *SlILL1*, *5*, and *6* genes play major roles in mediating tomato abscission.

## Results

### Identification, isolation, and sequence analysis of the *SlILL* gene family in tomato

To identify the tomato *ILL* family genes, BLAST searches of the tomato sequences at the SGN database were performed using the *ILL* domains of the seven Arabidopsis proteins as queries. A total of seven *ILL*-domain-containing unigenes were obtained using TBLASTN searches. The full-length cDNA sequences of all seven putative *SlILL*s were isolated using PCR-based approaches. The lengths of the open reading frames (ORFs) of the *SlILL* genes varied from 1628 bp (*SlILL*2) to 1902 bp (*SlILL*7), and they are predicted to encode polypeptides of 430–485 aa. These genes were named *Solanum lycopersicum IAA-amino acid hydrolase/IAA-leucine resistant-like* (*SlILL*) genes (Table 1).

**Table 1:** Characteristics of the *ILL* gene family members from tomato (*Solanum lycopersicum*)

Gene name	Gene number in Sol Genomics database	Open reading frame length	Predicted polypeptide length	Cleavage site prediction (amino acid residues)	pI	kD Molecular weight	M20 dimerisation domain	HDEL sequence
*SlILL1*	Solyc01g005490.2.1	1818	439	Between 20 and 21	5.7	48.21	+	–
*SlILL2*	Solyc05g006220.2.1	1628	444	Between 21 and 22	6.22	49.08	+	–
*SlILL3*	Solyc06g054410.2.1	1899	438	Between 20 and 21	5.83	48.48	+	–
*SlILL4*	Solyc12g008690.1.1	1937	446	None	6.07	48.86	+	+
*SlILL5*	Solyc06g073060.2.1	1671	430	Between 21 and 22	5.7	47.38	+	–
*SlILL6*	Solyc10g079640.1.1	1902	485	None	7.7	53.52	+	–
*SlILL7*	Solyc03g121270.2.1	1718	445	Between 23 and 24	5.25	48.75	+	+

**Fig. 1:**
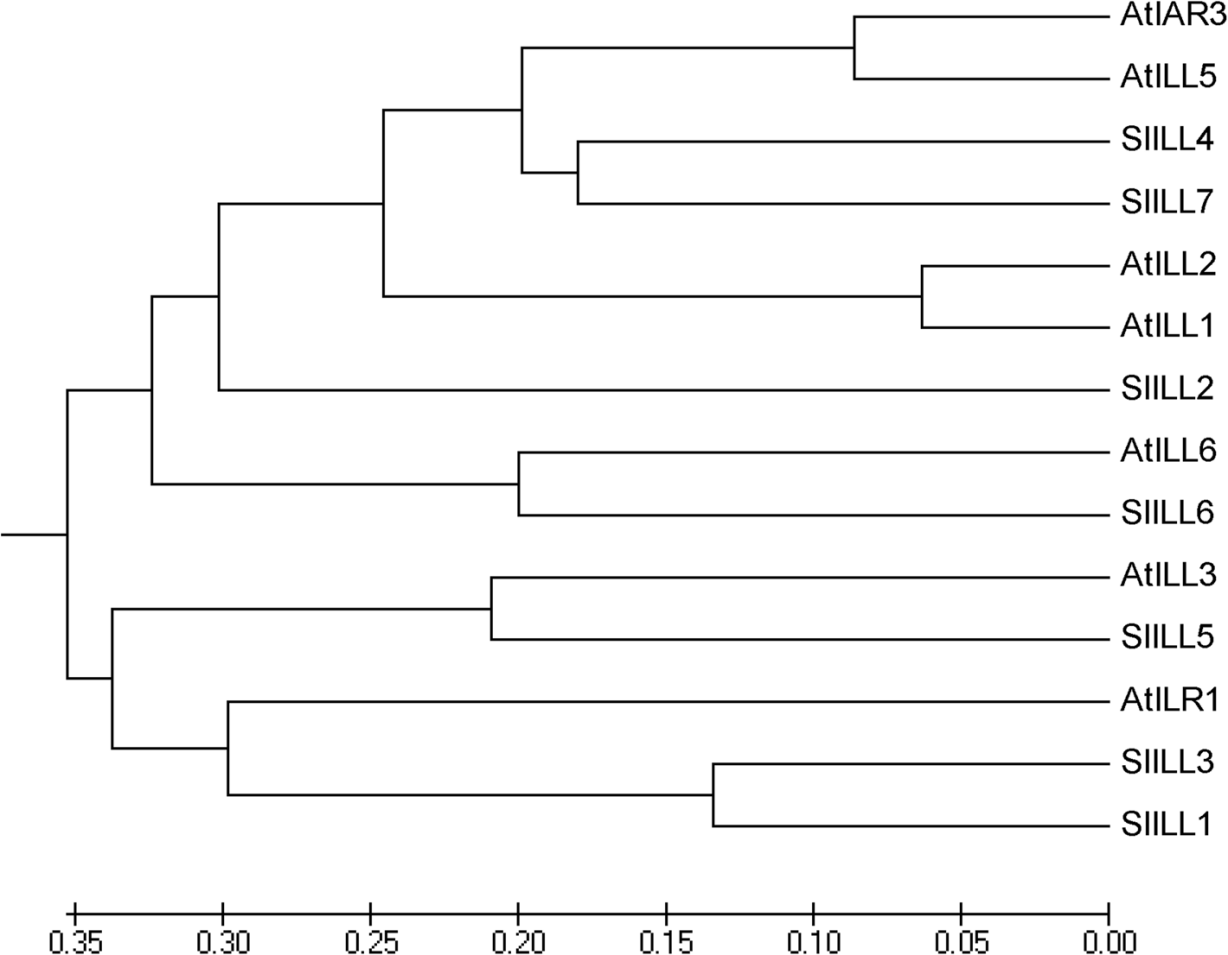
Phylogenetic analysis of tomato and Arabidopsis ILL proteins. The tree depicts the evolutionary relationships between ILL protein sequences of tomato (SlILLs) and Arabidopsis (AtILLs) The unrooted tree was generated using the neighbor-joining (NJ) method in MEGA4.1 and viewed in Fig Tree v1.2.2. The scale bar represents 0.06 amino acid substitutions per site

We used the PSORT program (http://www.genscript.com/psort.html) to predict protein localization sites in cells; analyses of the predicted protein products suggested that none of the hydrolases are localized to the endoplasmic reticulum (ER). The SlILL4 and SlILL7 proteins contain the N terminal HDEL sequence that has been annotated as an ER retention signal, but according to PSORT, the probability of ER localization is low. The sequences of SlILL4 and SlILL6 appear to possess an uncleavable N-terminal signal sequence, while the other SlILL proteins all have a cleavable N-terminal signal sequence, after which the mature proteins are predicted to start (Table 1). The percent identity between the different tomato and Arabidopsis ILR1-like proteins ranges between 44 and 87% (Additional file 1: Figure S1, Table 2). Phylogenetic analysis of the predicted ILL protein sequences revealed that the AtILR and SlILL proteins are divided into two well defined clades; SlILL2, 4, 6, and 7 are in cluster I, and SlILL 4 and SlILL7 show a close relationship to AtIAR3 and AtILL5, while SlILL6 clusters with AtILL6. SlILL1, 3, and 5 are in cluster II, with SlILL1 and 3 in a subcluster with AtILR1, and SlILL5 clusters with AtILL3 (Fig. 1).

**Table 2:** Pair-wise percent identities between IAA conjugate hydrolase sequences from *Solanum lycopersicum* (Sl) and *Arabidopsis thaliana* (At)

Similarity (%)	SlILL1	SlILL2	SlILL3	SlILL4	SlILL5	SlILL6	SlILL7	AtILR1	AtIAR3	AtILL1	AtILL2	AtILL3	AtILL5	AtILL6
SlILL1		52	75	54	56	54	52	58	55	54	54	53	55	52
SlILL2			47	57	51	56	57	51	55	58	57	50	55	54
SlILL3				53	54	50	50	55	48	51	50	51	49	49
SlILL4					53	55	68	48	66	64	65	48	62	53
SlILL5						54	56	54	52	51	53	64	51	53
SlILL6							56	49	55	54	54	49	54	68
SlILL7								49	67	61	63	48	64	55
AtILR1									49	47	50	49	46	49
AtIAR3										60	62	48	83	50
AtILL1											88	46	58	53
AtILL2												49	59	51
AtILL3													48	51
AtILL5														50
AtILL6

### Auxin conjugate hydrolase activity of the SlILL proteins

The SlILL proteins were purified using affinity chromatography (Additional file 2: Figure S2) and tested for IAA conjugate hydrolysis activity using different IAA amino acid conjugates. All SlILLs hydrolyzed IAA-Ala efficiently, and SlILL1 had the highest activity. Similar to IAA-Ala, all of the SlILLs were able to hydrolyze IAA-Ile and IAA-Gly, but had lower activities against these substrates. Only SlILL1 and SlILL4 hydrolyzed IAA-Asp, and the relative activities were very low. None of the SlILLs could hydrolyze IAA-Trp. (Table 3).

**Table 3:** Auxin conjugate hydrolase activity analysis of purified His-tagged SlILL proteins. Values shown are the means of three time points ± S.D. The detection limit was 1 nmol IAA released mg^−1^ min^−1^

Substrate	SlILL1	SlILL2	SlILL3	SlILL4	SlILL5	SlILL6	SlILL7
IAA-Ala	62.3 ± 15.1	31.5 ± 6.2	36.2 ± 12.1	24.5 ± 15.3	23.6 ± 8.2	45.1 ± 14.5	39.2 ± 15.1
IAA-Ile	4.5 ± 1.3	2.3 ± 1.3	3.2 ± 2.6	3.6 ± 2.2	4.6 ± 2.1	4.3 ± 1.6	3.5 ± 1.9
IAA-Asp	3.5 ± 3.1	< 1	< 1	2.2 ± 1.8	< 1	< 1	< 1
IAA-Gly	14.2 ± 4.2	4.4 ± 1.3	5.2 ± 2.1	4.6 ± 1.7	6.4 ± 2.3	8.7 ± 3.2	8.2 ± 2.6
IAA-Trp	< 1	< 1	< 1	< 1	< 1	< 1	< 1

### Expression patterns of *SlILL* genes in different organs of tomato

**Fig. 2:**
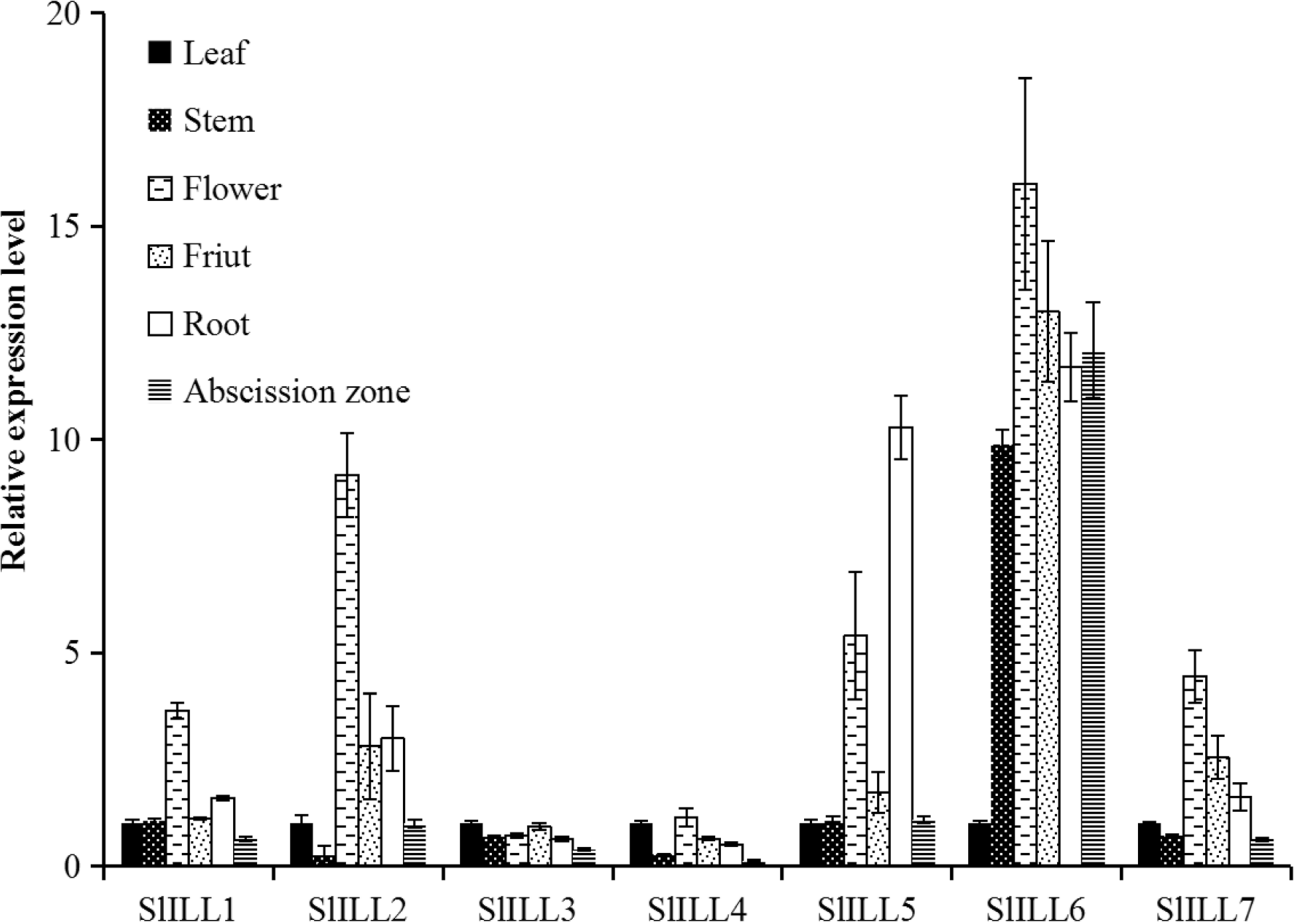
Expression profiles of the *SlILL* genes analyzed during tomato development: (filled bar) leaf; (high density dot) stem; (broken line) flower; (low density dot) fruit; (empty bar) root; (horizontal hatching) AZ. The quantitative PCR data represent mean values for three independent biological replicates ± SD

The *SlILL* gene expression patterns in several tomato organs were also investigated. *SlILL1* showed its highest level of expression in the flower, and the lowest expression level in the abscission zone, with intermediate levels of expression in the leaf, stem, and fruit. *SlILL2* was most highly expressed in the flower, fruit, and AZ, while extremely low levels of expression were found in the stem. For *SlILL3*, a similar expression level was found in all tomato organs except for low expression in the AZ. *SlILL4* was expressed at a low level in the stem and AZ, while expression was somewhat higher in the other organs. The highest expression levels of *SlILL5* were in the flower and root, with lower levels of expression in the other organs. *SlILL6* was expressed at high levels in all organs except the leaf, and *SlILL7* had relatively high levels of expression in the flower, fruit, and root (Fig. 2).

### The effects of different auxin conjugates on abscission

**Fig. 3:**
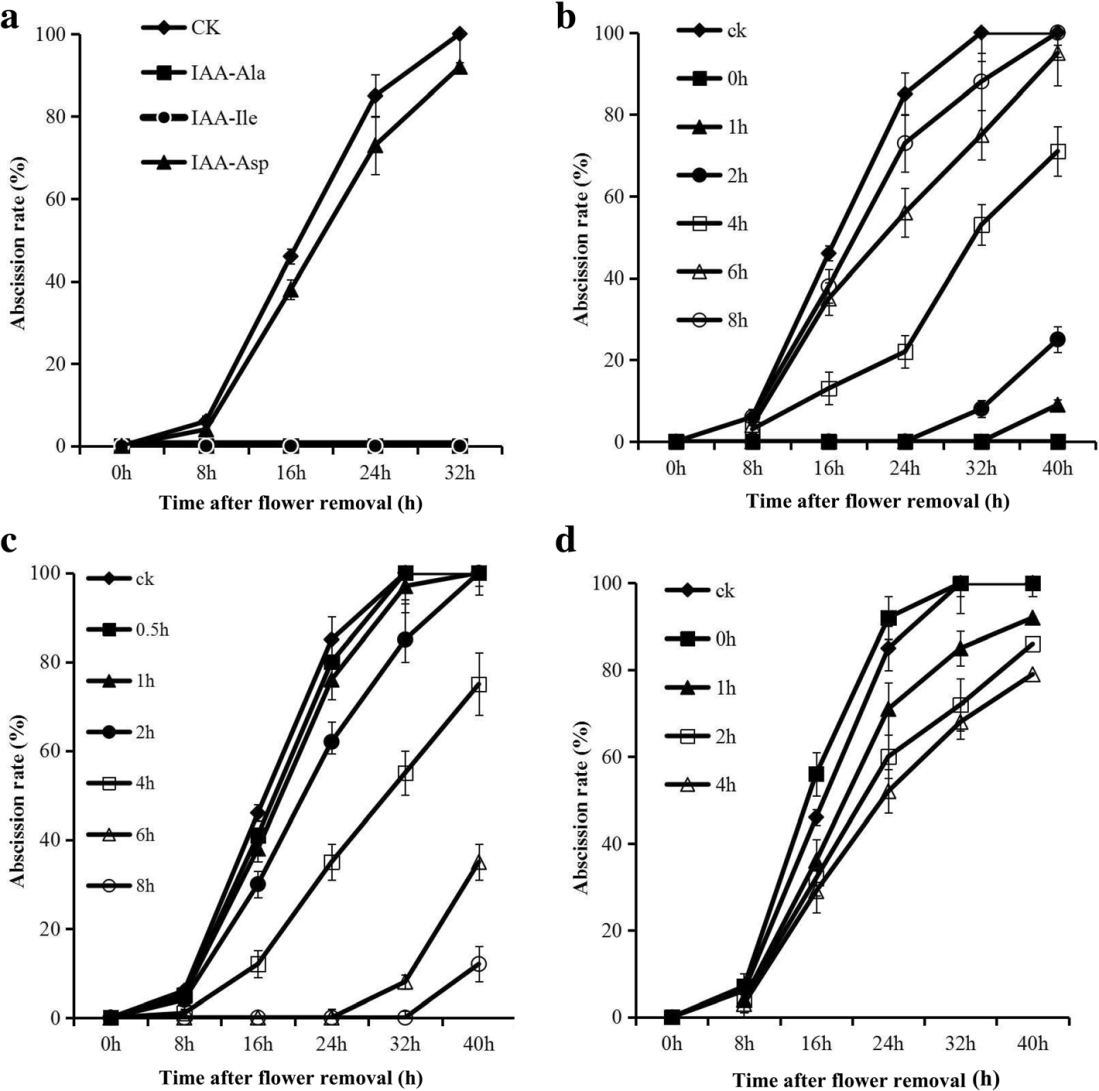
(**a**) Effects of exogenous IAA-Ala, IAA-Ile, and IAA-Asp on the abscission rate of tomato pedicel explants: (filled diamond) control (CK); (filled square) 30 mM IAA-Ala treatment; (filled circle) 30 mM IAA-Ile treatment; (filled triangle) 30 mM IAA-Asp treatment. **b** Flower pedicels were pre-incubated in MS medium and then incubated in IAA-Ile at different times for abscission analysis: (filled diamond) control (CK); (filled square) incubated in IAA-Ile agar at 0 h; (filled triangle) 1 h; (filled circle) 2 h; (empty square) 4 h; (empty triangle) 6 h; (empty circle) 8 h. **c** Flower pedicels were pre-incubated in IAA-Ile for different times and then transferred to MS medium for abscission analysis: (filled diamond) control (CK); (filled square) incubated in 30 mM IAA-Ile agar for 0.5 h; (filled triangle) 1 h; (filled circle) 2 h; (empty square) 4 h; (empty triangle) 6 h; (empty circle) 8 h. **d** Flower pedicels were pre-incubated in MS and then incubated in IAA-Ile with cycloheximide (CHX) at different times for abscission analysis: (filled diamond) control (CK); (filled square) incubated in 30 mM IAA-Ile with CHX agar at 0 h; (filled triangle) incubated in 30 mM IAA-Ile with CHX agar at 1 h; (empty square) 2 h; (empty triangle) 4 h. All results are the means of three replicates (60 flowers each) ± SE

To determine the effect of auxin conjugates on abscission, 30 μM solutions of IAA-Ala, IAA-Asp, and IAA-Ile were applied to pedicel explants. Both IAA-Ala and IAA-Ile could significantly delay abscission, while IAA-Asp had little effect. IAA-Ala has free auxin activity without hydrolysis; therefore, IAA-Ile was used for the subsequent abscission assays (Fig. 3a).

To explore the optimal time of auxin conjugates application for abscission, two experiments were performed. First, explants were pre-incubated on control agar (without IAA-Ile) then transferred to agar containing IAA-Ile. This experiment showed that explants incubated on control agar and transferred to IAA-Ile agar before 6 h showed a significant level of abscission. However, there was no significant inhibition of abscission after 6 h (Fig. 3b). Second, the explants were initially pre-incubated for different times in IAA-Ile and then transferred to agar without IAA-Ile. The results indicated that only 2 h of pre-incubation with IAA-Ile was sufficient to inhibit abscission. (Fig. 3b, c).

We performed another experiment to test whether there is adequate IAA-amino acid hydrolase activity in the AZ to inhibit abscission if there is an adequate level auxin conjugate present. The explants were incubated with or without CHX for 0, 2, and 4 h, and then transferred to IAA-Ile agar containing CHX. The results indicated that CHX could accelerate abscission compared with the control and drastically reduced the inhibition of abscission by IAA-Ile. Notably, the 0-h CHX treatment shows a higher rate of abscission than the control (Fig. 3d).

### Auxin conjugates can modify the free IAA concentration in the AZ

**Fig. 4:**
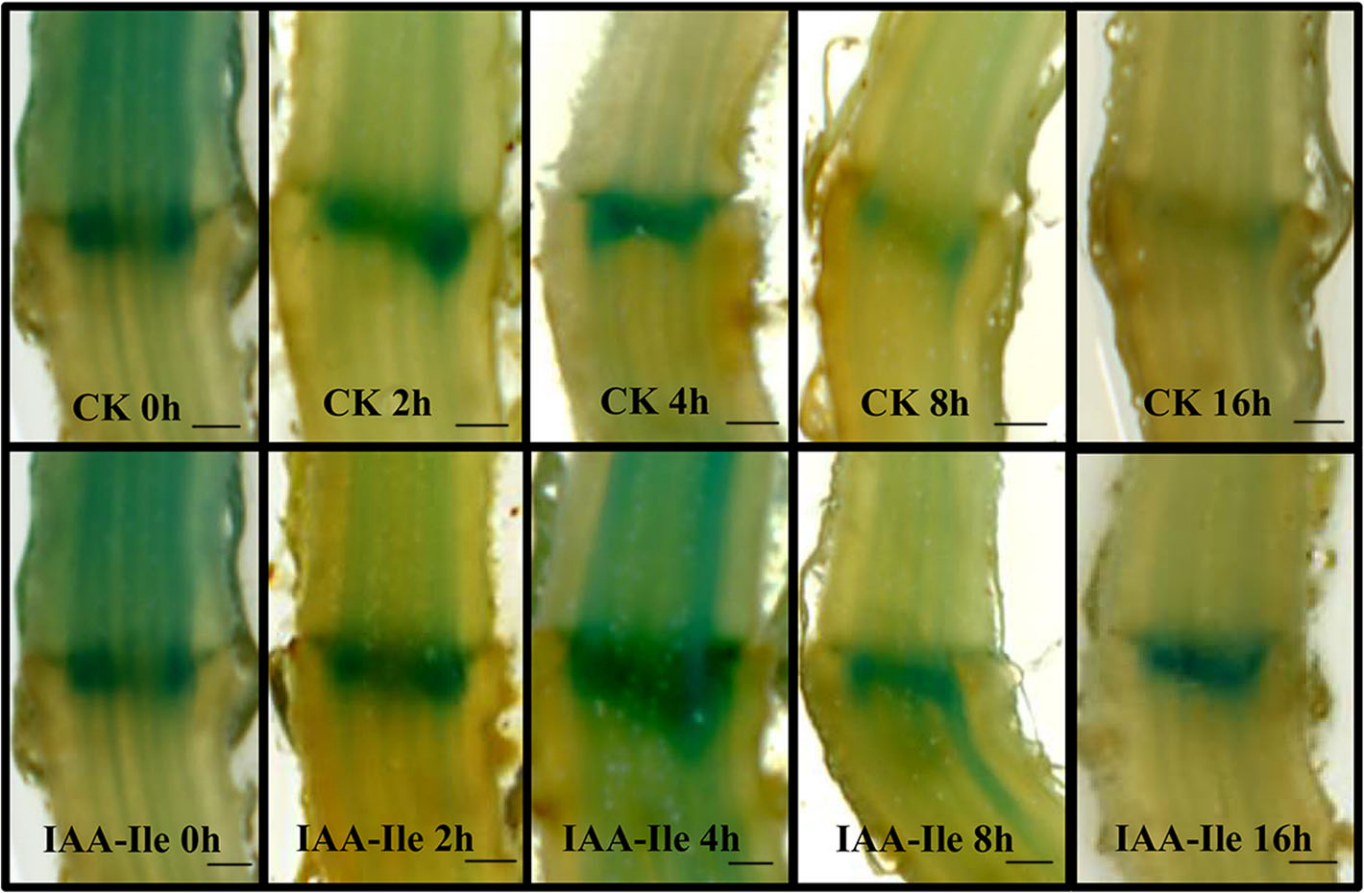
The effects of IAA-Ile on auxin distribution during abscission in tomato pedicel explants. Scale bars = 0.3 mm

**Fig. 5:**
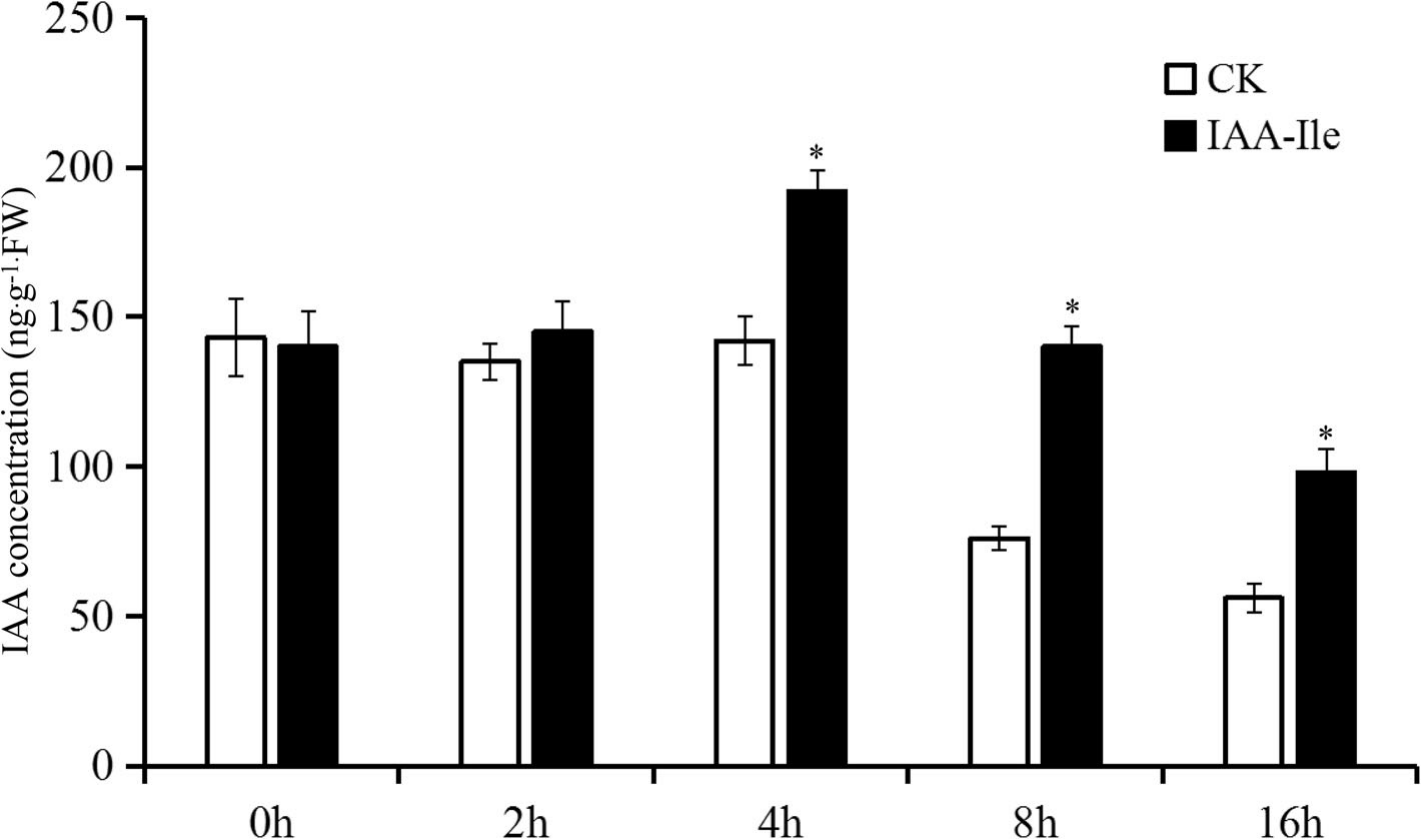
The effect of IAA-Ile on auxin concentration in AZ during abscission

After removal of flowers from the auxin reporter DR5::GUS lines, β-Glucuronidase (GUS) staining in the AZ was stable before 2 h, increased at 4 h, and then decreased to 16 h. Incubation with IAA-Ile could significantly increase the auxin content in the AZ, and the significant decrease observed after 4 h showed that the auxin conjugate could enhance free auxin concentration in the AZ. It is notable that there was no significant difference in GUS activity at 2 h in the AZ between the control and the IAA-Ile treatment (Fig. 4). There was adequate auxin conjugate (as 4 h could release more free IAA) present in the AZ and IAA-Ile in the agar; therefore, the only reason for this phenomenon is insufficient auxin conjugate hydrolysis in the AZ before 2 h. Auxin concentration analysis confirmed that IAA-Ile could significantly enhance the AZ auxin concentration after 4 h compared with that in the control (Fig. 5, Additional files 3 and 4). Moreover, in the IAA-Ile treatment, the free auxin did not show a constantly increasing trend, but rather showed a slight decrease after 4 h.

### Expression of *SlILL* genes in response to 1-MCP and auxin treatment during flower-removal pedicel abscission

**Fig. 6:**
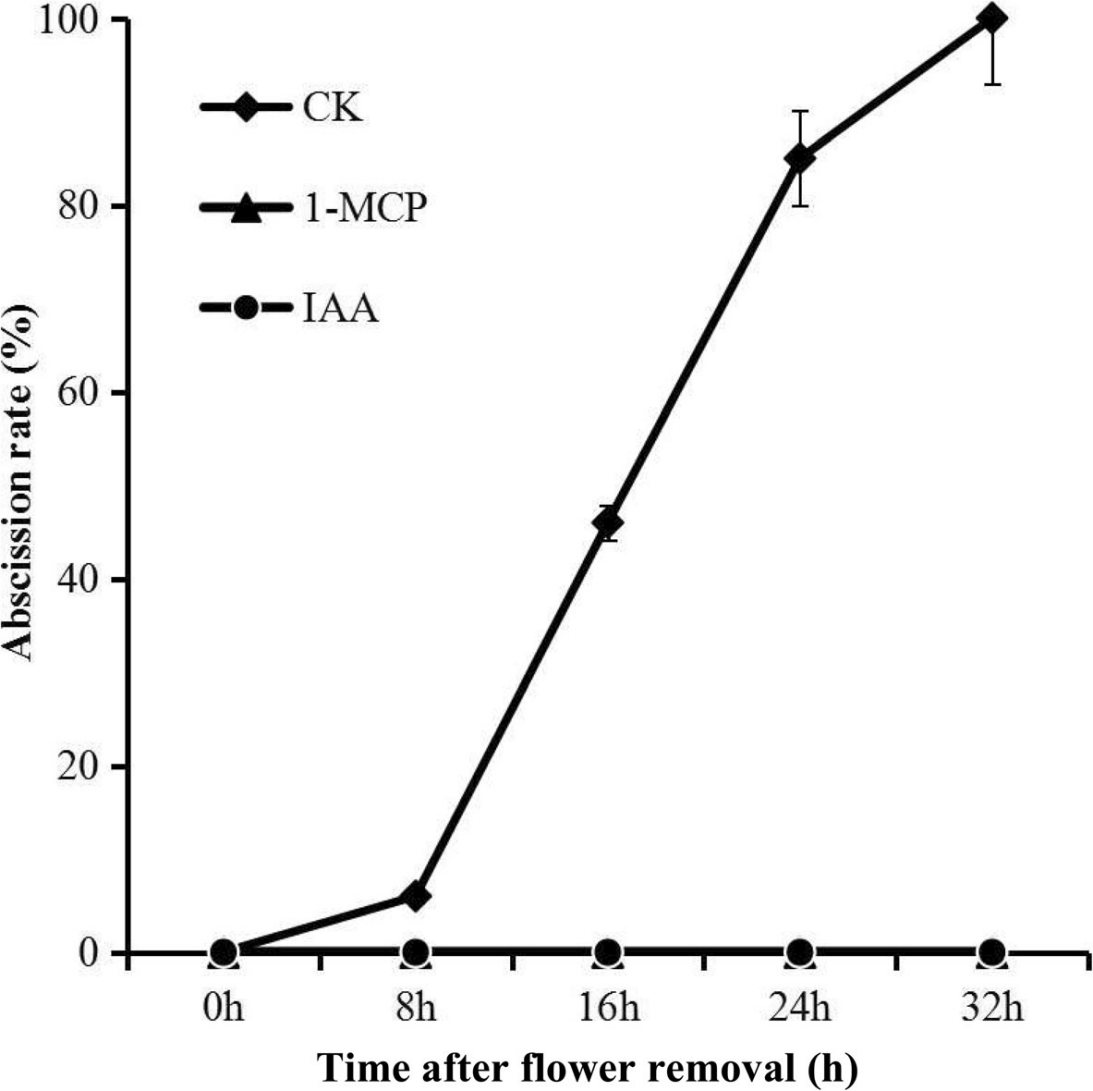
Effects of 1-MCP and auxin on the abscission rate of tomato pedicel explants: (filled diamond) control (CK); (filled circle) 50 μg·g^− 1^ auxin treatment; (filled triangle) 20 μl·l^− 1^ 1-MCP treatment. The results are means of three replicates (60 flowers each) ± SE. The agar medium contained deionized water (control) or was supplemented with 50 μg·g^− 1^ IAA. For the 1-MCP treatment, 1-MCP (20 μl·l^− 1^) was applied for 24 h to treat the whole plant before the tomato flower was removed and incubated in agar

**Fig. 7:**
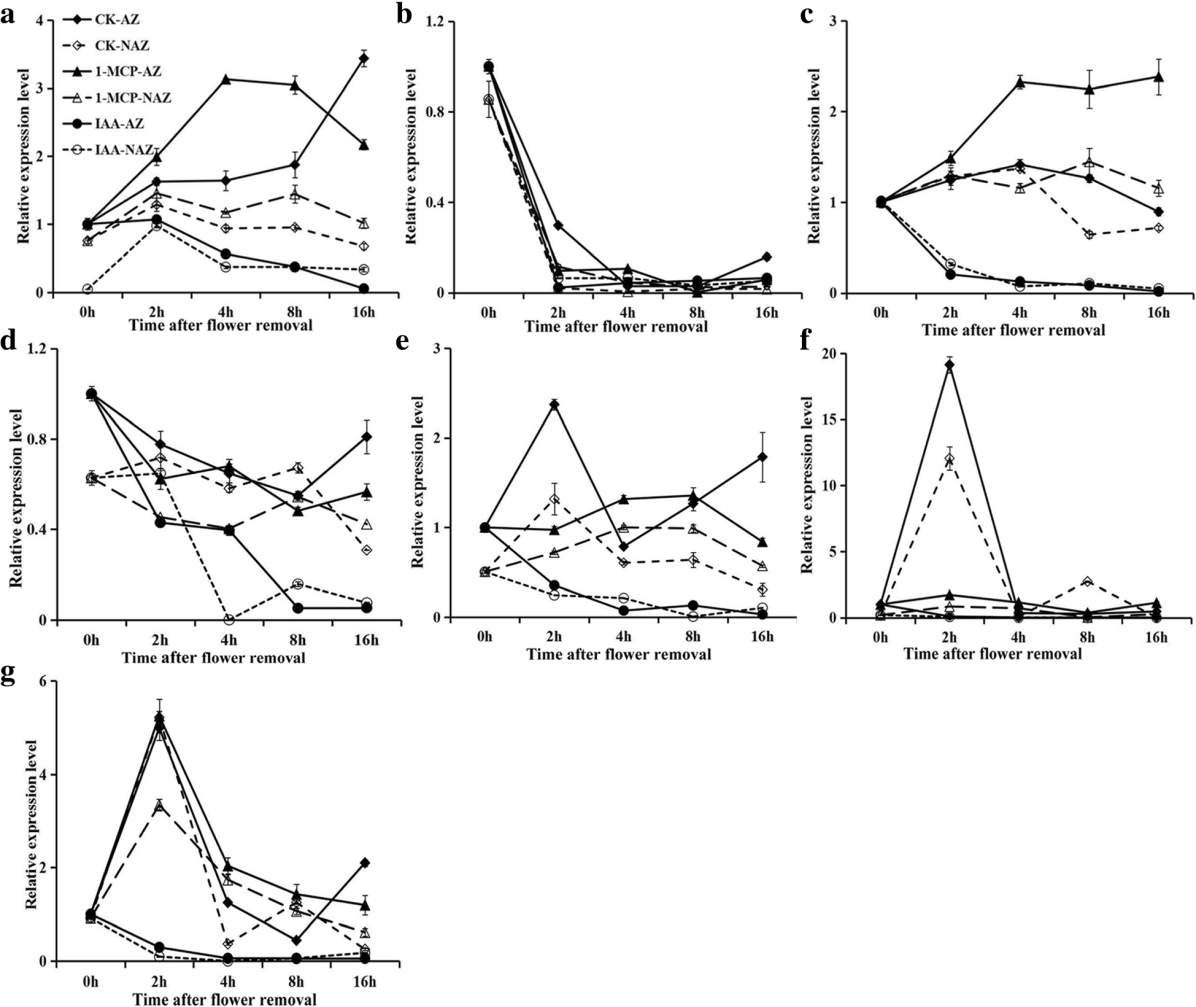
Expression profiles of the seven *SlILL* genes in response to different treatments during pedicel abscission. *SlILL1* (**a**), *SlILL2* (**b**), *SlILL3* (**c**), *SlILL 4*(**d**), *SlILL5* (**e**), *SlILL6* (**f**), and *SlILL7* (**g**): (filled diamond) control abscission zone (CK-AZ); (filled triangle) 1-MCP treated abscission zone (1-MCP-AZ); (filled circle) IAA treated abscission zone (IAA-AZ); (empty diamond) control proximal part (CK-NAZ); (empty triangle) 1-MCP-treated proximal part (1-MCP-NAZ); (empty circle) IAA-treated proximal part (IAA-NAZ). The results are means of three biological replicates and three technical replicates ± SD

Abscission rates (AR) of tomato pedicel explants increased significantly after 8 h, reaching 100% at 32 h, while auxin or 1-MCP (1-methylcylopropene, a competitive inhibitor of ethylene action that binds to the ethylene receptor to regulate tissue responses to ethylene) completely inhibited abscission before 48 h (Fig. 6). *SlILL1* showed steadily increasing expression in the control AZ during abscission, while its expression remained relatively stable in the NAZ (the proximal part of the AZ). 1-MCP treatment increased the expression of this gene in both the AZ and NAZ. Unlike *SlILL1*, *SlILL2* expression decreased sharply immediately after flower removal in both the AZ and NAZ. 1-MCP treatment did not alter *SlILL2* expression. *SlILL3* expression showed a slight increase in the control (CK) up to 4 h, followed by a decrease in the AZ, and a similar expression pattern was found in the NAZ. 1-MCP enhanced *SlILL3* expression in both the AZ and, to a lesser extent, in the NAZ. *SlILL4* showed decreased expression until 8 h, after which expression increased in the AZ, while expression was relatively stable until 8 h in the NAZ, after which it decreased. *SlILL5* showed a ~ 2.5-fold increase in expression at 2 h, followed by a decrease at 4 h to below the 0-h level, then an increase of ~ 2-fold by 16 h in the AZ, while expression increased at 2 h then decreased steadily until 16 h in the NAZ. 1-MCP treatment inhibited *SlILL5* expression before 2 h in the AZ and increased expression at 4 h and 8 h in the AZ and NAZ. *SlILL6* expression increased 20-fold at 2 h then decreased to a very low level in both the AZ and NAZ. 1-MCP significantly inhibited the expression of *SlILL6* in the AZ and NAZ. Expression of *SlILL7* showed a significant increase at 2 h, and then decreased both in the AZ and NAZ. 1-MCP had little effect on its expression in the AZ, while a slight increase in expression was seen in the NAZ. Auxin treatment could significantly inhibit gene expression for all *SlILL*s in both the AZ and NAZ during abscission. Overall, the expression levels of *SlILL1, 5*, and *6* showed a continuous increase in the AZ, which is distinct from the NAZ, and was classified as AZ special expression; the expression of these genes was affected by 1-MCP, and they were selected as abscission-related auxin conjugate hydrolase genes for further study (Fig. 7).

### Accumulation of SlILLs protein in response to 1-MCP treatment during flower-removal pedicel abscission

**Fig. 8:**
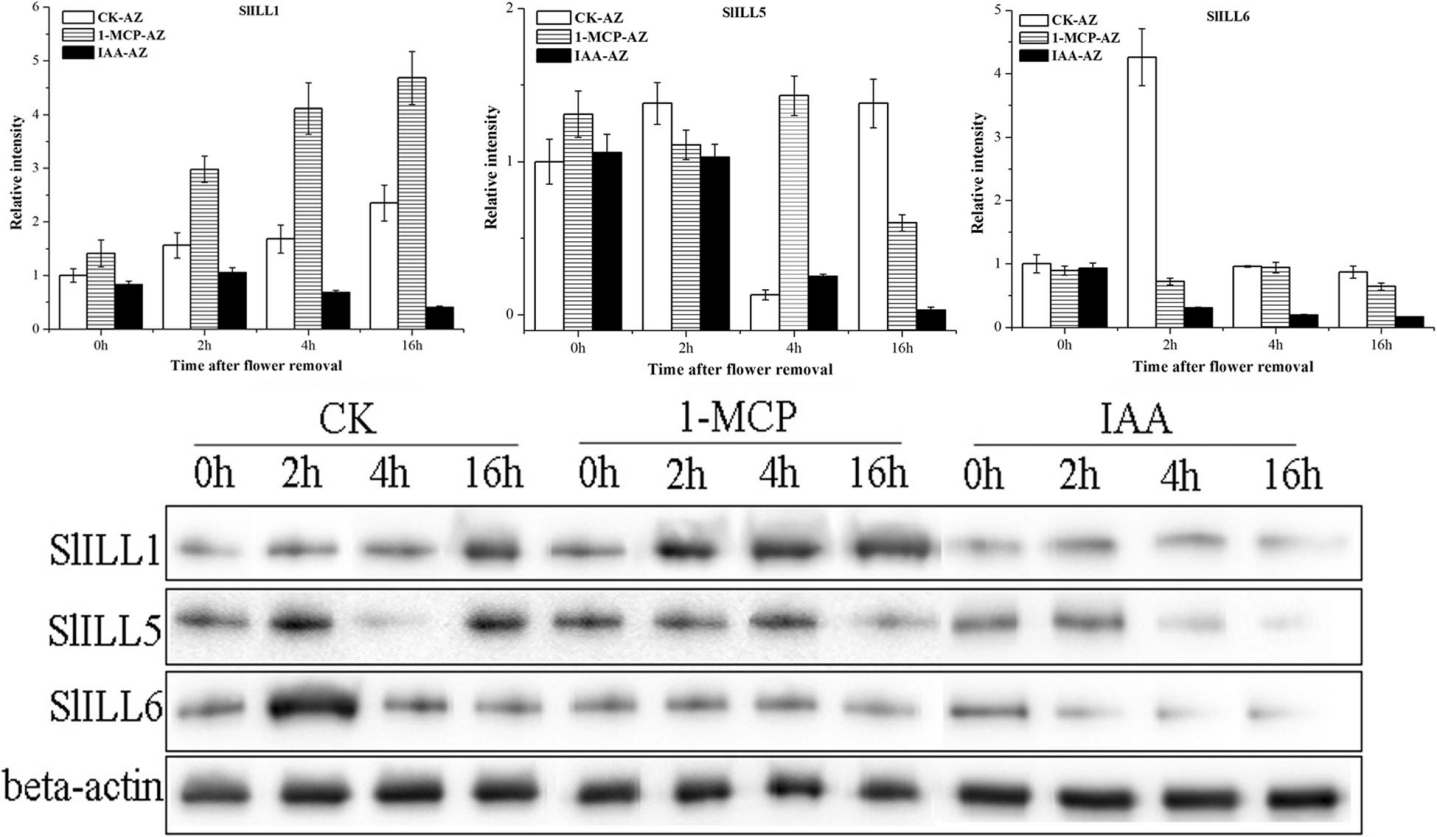
Accumulation of SlILL1, SlILL5, and SlILL6 proteins in response to 1-MCP and IAA treatment during abscission

SlILLs protein accumulation in the AZ was then assayed. The level of SlILL1 protein increased steadily during abscission, and 1-MCP treatment enhanced the accumulation of this protein. The level of SlILL5 increased at 2 h, then decreased at 4 h and increased sharply at 16 h, while 1-MCP treatment inhibited the level of SlILL5 protein before 2 h, increased the level at 4 h, and decreased its accumulation at 16 h. The level of SlILL6 reached a peak at 2 h and then decreased and maintain a level until 16 h, 1-MCP could significantly inhibit the accumulation of SlILL6 at 2 h. IAA treatment significantly depressed SlILL1, SlILL5, and SlILL6 translation throughout abscission (Fig. 8).

### Using VIGS to downregulate *SlILL1, SlILL5*, and *SlILL6* gene expression significantly accelerated pedicel abscission

To understand the function of *SlILL* genes in abscission, the effect of silencing *SlILLs* on pedicel abscission was examined using VIGS. In this study, we used ten silencing constructs designed to silence *SlILL1, SlILL3, SlILL5, SlILL6*, and SlILL7 individually, to double-silence *SlILL1 + 5*, *SlILL1 + 6*, *SlILL5 + 6*, and *SlILL3 + 7*, and to triple silence *SlILL1 + 5 + 6* in tomato. The *SlILL* gene expression levels in the different *SlILL* VIGS lines were quantified using qRT-PCR. The results confirmed the downregulation of the target *SlILLs* without affecting expression of the other *SlILL* genes.

**Fig. 9:**
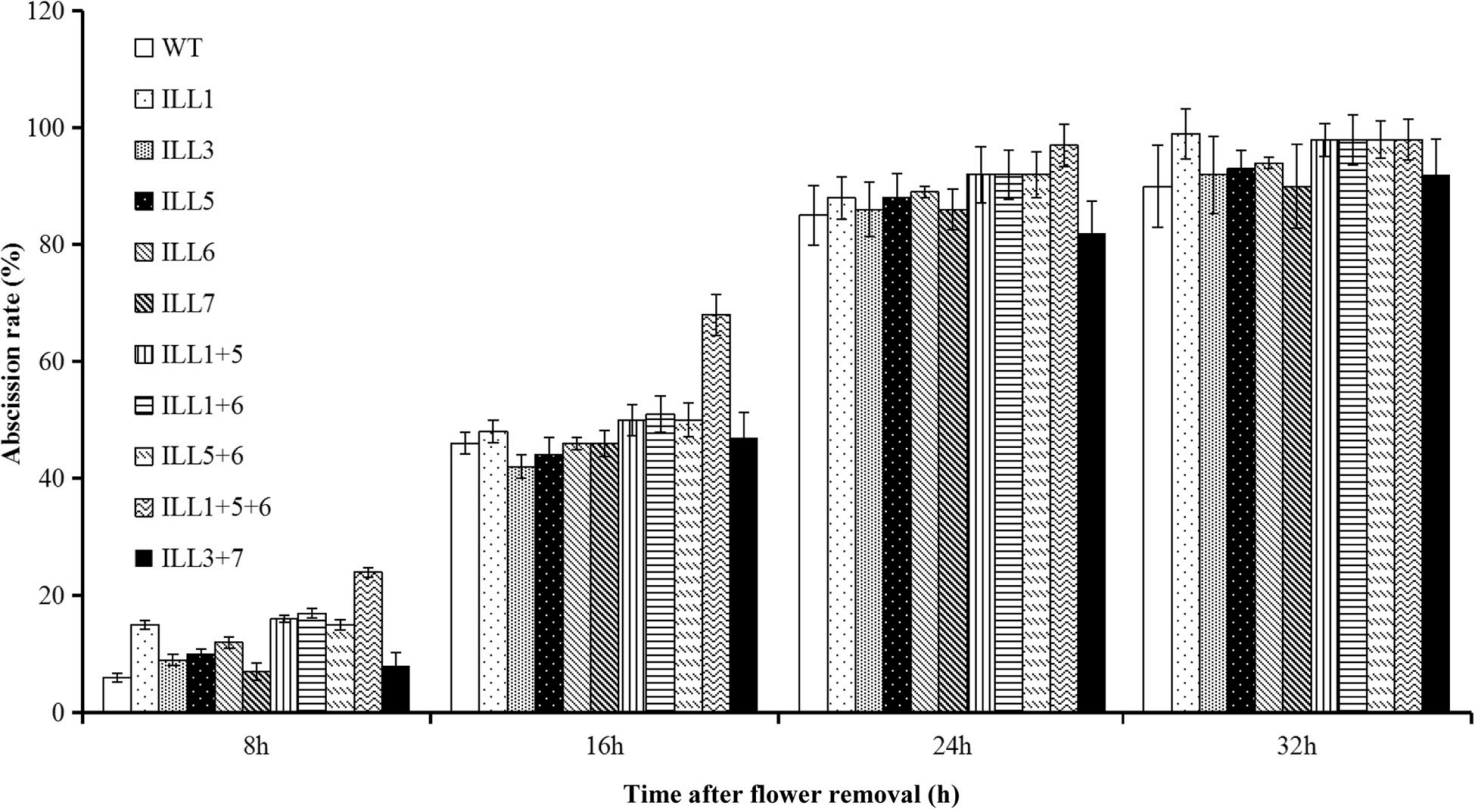
Analysis of pedicel abscission in tomato plants in which the expression of the *SlILL* genes was downregulated using virus-induced silencing (VIGS). The abscission rates of pedicels were determined at different times after flower removal: (empty bar) wild-type control (CK); (low density dot bar) silencing the expression of *SlILL1*; (high density dot bar) silencing of *SlILL3*; (dark bar with white dots) silencing of *SlILL5*; (slash line bar) silencing of *SlILL6*; (dark slash line bar) silencing of *SlILL7*; (vertical line bar) double silencing of *SlILL1* and *SlILL5*; (horizontal line bar) double silencing of *SlILL1* and *SlILL6*; (broken slash line bar) double silencing of *SlILL5* and *SlILL6*; (wavy line bar) silencing of *SlILL1*, *SlILL5*, and *SlILL6*; and (black bar) double silencing of *SlILL3* and *SlILL7*. * indicates significant differences at *P* < 0.05. Results are the means of three replicates ± SD, with at least 15 samples per replicate

In the control, the abscission rate was 43% at 16 h, 85% at 24 h, and 100% at 32 h. Silencing *SlILL1*, *SlILL5*, and *SlILL6* individually significantly accelerated abscission, with 54% pedicel explants abscised at 16 h and 93% at 24 h. The double-silenced *SlILL1 + 5, SlILL1 + 6, SlILL5 + 6* lines all showed increased abscission > 60% at 16 h and 100% at 24 h; however, the *SlILL3 + 7* double-silenced line showed no significant difference compared with the control*.* The triple-silenced *SlILL1 + 5 + 6* line showed the most obviously increased abscission rate, which was higher than any of the double-silenced *SlILL* lines (Fig. 9)*.* The time required for 50% abscission also indicated that *SlILL1, SlILL5*, and *SlILL6* have important and cooperative roles in mediating abscission in tomato (Table 4).

**Table 4:** Time required (in hours) for the different VIGS ILL lines to reach 50% flower pedicel abscission

Silenced *SlILL* gene(s)	CK	ILL1	ILL3	ILL5	ILL6	ILL7
Time to 50% abscission	17.8 ± 0.29 a	16 ± 0.32 b	17.5 ± 0.32 a	16.3 ± 0.29 b	16.2 ± 0.22 b	17.7 ± 0.16 a
Silenced *SlILL* gene(s)	ILL1 + 5	ILL1 + 6	ILL5 + 6	ILL3 + 7	ILL1 + 5 + 6	
Time to 50% abscission	15.3 ± 0.41 c	15.4 ± 0.19 c	15.3 ± 0.35 c	17.6 0.53 a	12.4 ± 0.33 d	

a, b, c, and d indicate significant differences at P < 0.05. CK as the control. Results are the means of three replicates ± SD, with at least 15 samples per replicate

### Downregulation of *SlILL1*, *5*, and *6* significantly altered auxin concentration and polar auxin transporter (*PIN*) gene expression in the AZ

**Fig. 10:**
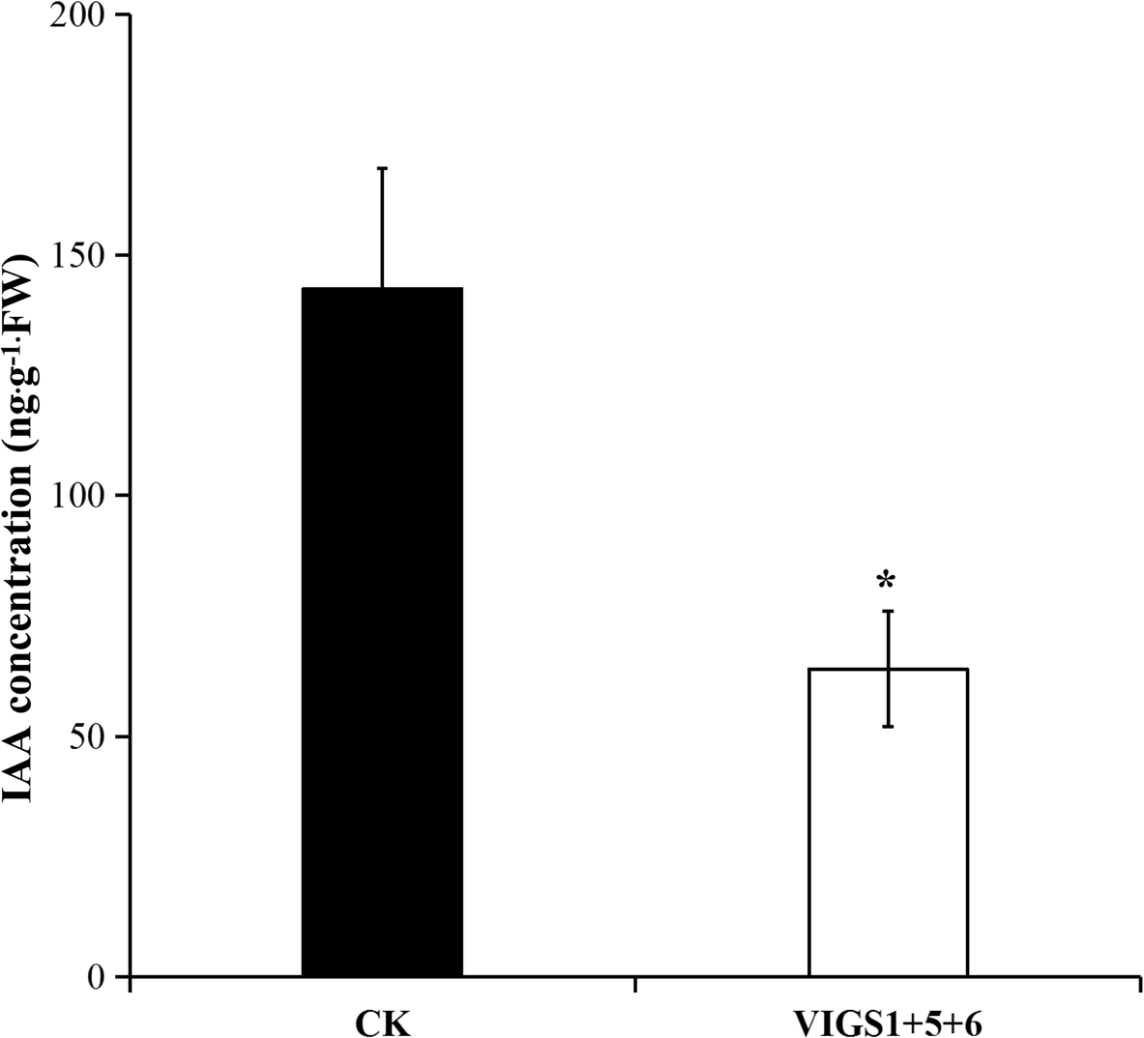
The effect of downregulated expression of *SlILL1*, *SlILL5*, and *SlILL6* on the auxin concentration in AZ after flower removal 4 h. The results are means of three biological replicates and three technical replicates ± SD

**Fig. 11:**
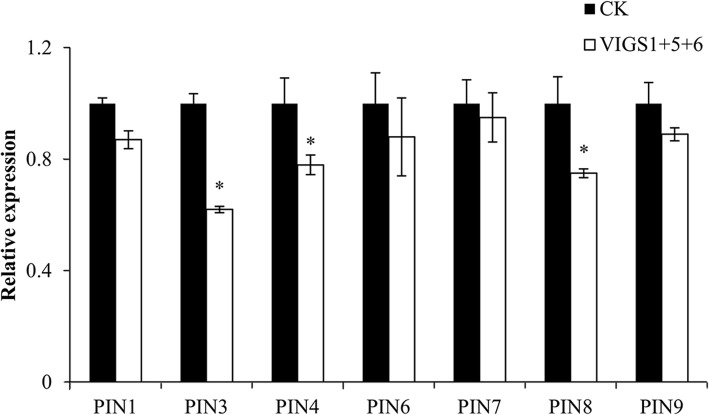
The effects of downregulated expression of *SlILL1*, *SlILL5*, and *SlILL6* on the expression of seven *PIN* genes in AZ during abscission. The results are means of three biological replicates and three technical replicates ± SD

*SlILL5* and *6* showed peak expression at 2 h, and *SlILL1* expression increased before 4 h in the AZ. Combined with the slight IAA concentration increase in the AZ at 4 h, we evaluated the effects of downregulating *SlILL1 + 5 + 6* expression on the auxin concentration and *PIN* gene expression. The AZ was sampled from VIGS *SlILL1 + 5 + 6* lines, which showed the most obviously increased abscission rate. The results showed that the auxin content was significantly decreased in the VIGS triple-silenced *SlILL1 + 5 + 6* lines compared with that in the control (Fig. 10, Additional files 3 and 4). In addition, we checked the expression levels of all PINs in the AZ, and only PIN1, 3, 4, 6, 7, 8, and 9 have expression in AZ, and the expression of *PIN3*, *4*, and *8* was significantly decreased in these lines (Fig. 11).

## Discussion

Auxin conjugation and hydrolysis play important roles in mediating the auxin balance for proper plant development and response to environment signals [2, 23]. Several auxin conjugate hydrolases have been isolated from the dicots *Brassica rapa, Medicago truncatula*, and *Arabidopsis thaliana* and the monocots *Triticum aestivum* and rice, and are involved in root development, pathogenesis, and grain weight [9, 16, 24–26]. The biochemical characterization of substrate specificity and the different temporal and spatial gene expression patterns further revealed the complex role of IAA conjugate hydrolases in auxin signaling [25, 27]. Although our understanding of IAA-regulated abscission processes, including the molecular mechanisms of action, perception, and transport, comes from studies of gene transcription analyzed in pre-abscission or auxin-depleted induced abscission, there remains a lack of knowledge about the potential role of IAA conjugate hydrolases in mediating abscission [20, 22, 28].

### Identification, sequencing, and catalytic activity analysis of *ILL* genes and proteins in tomato

In *Arabidopsis*, seven genes of the amidohydrolase family have been well characterized: ILR1, ILL1, ILL2, and IAR3 can cleave IAA amino acid conjugates, while ILL3 and ILL6 cannot, and *ILL5* is thought to be a pseudo gene [11, 26, 29]. Genome scanning and DNA sequencing identified seven full-length tomato cDNAs, *SlILL1* through *SlILL7*, that are highly homologous to the IAA-amidohydrolases of *Arabidopsis*. The IAR3-like (*Arabidopsis* and *Brassica*) AtILL1 and AtILL5 proteins contain a C-terminal KDEL sequence that signals retention of plant proteins in the lumen of the ER. However, none of the SlILL proteins contains a terminal KDEL, and are therefore predicted to have a lower probability of being located on the ER, although SlILL1, 2, and 7 have a terminal HDEL motif (similar to Br-ILL6), which is annotated by PSORT as an ER retention signal [15]. The importance of ER localization is not clear, however, because phylogenetic analysis of 66 orthologs from across the plant kingdom resolved two separate monocot clades, one with members possessing ER retrieval sequences and one lacking them [2, 30, 31]. The ER retention signal is not required for activity, because two of the five amidohydrolases characterized from *M. truncatula* lack such a tetrapeptide, but still show activity [14], and TaIAR3 has the unusual C-terminal sequence motif RDEL [16]. Moreover, a cleavable N-terminal signal sequence was found in SlILL1, 2, 3, 4, and 7, while SlILL5 and 6 contain an uncleavable N-terminal sequence.

Previously, the structure of AtILL2 was found to be similar to members of the M20 peptidase family, which suggests that ILL2 likely uses a catalytic mechanism similar to that established for the M20 peptidase enzymes family. All SlILLs have the M20 dimerization domain, which implies that all of them have auxin conjugate hydrolysis activity. The experiment further indicated that all SlILLs are able to hydrolyze IAA-Ala. The different enzymes also showed distinct patterns of hydrolase activity. AtILL2 preferentially catalyzes the hydrolysis of IAA-Ala and the hydrolytic activity is higher than that of SlILLs, while AtILL1, which shares 87% sequence identity with AtILL2, showed a lower activity than the SlILLs toward IAA-Ala. AtILR1 prefers IAA-Leu, IAA-Tyr, and IAA-Phe, while showing less activity toward IAA-Ile, and less activity than SlILLs. ILL3 and ILL6/GR1 have no such catalytic activity towards IAA conjugates [11, 30]. In *Triticum aestivum,* TaIAR3 has far less activity than SlILLs toward all of IAA-Ala, IAA-Ile, IAA-Asp and IAA-Gly substrates [4]. In *Medicago truncatula*, MtIAR31, − 32, − 33, and − 34 have hydrolytic activity toward IAA-aspartate and IBA-alanine, and shows higher activity than SlILLs toward IAA-Asp [16]. No hydrolase activity was found for IAA–Asp in vitro for all seven SlILLs; IAA–Asp may be an uncleavable auxin conjugate, and has no effect on abscission, hence it is possible that there is no specific auxin conjugate hydrolase for IAA-Asp in tomato.

### Auxin conjugates are involved in flower pedicel abscission

Cellular IAA is mainly present as amide derivatives and, to a lesser extent, as ester-linked conjugates, in *Arabidopsis thaliana* [32]. The most common auxin conjugates in Arabidopsis are IAA-Ala, IAA-Leu, IAA-Asp, IAA-Glu, IAA-glucose, and protein/peptide conjugates [23, 32–35]. Auxin conjugation is reversible through the actions of specific hydrolases, with the subsequent release of free IAA. Therefore, IAA conjugates have auxin activity when applied exogenously and show physiological activity in regulating different developmental processes, such as seed germination and root elongation.

The level of endogenous auxin must fall below a certain threshold in the abscission zone to initiate abscission [36, 37]. Endogenous auxin would be synthesized de novo and is mainly derived from young organs such as flowers, young fruits, and seeds; therefore, the local auxin concentration in the AZ is mainly mediated by auxin flux from flowers to stems. Flower removal abolishes auxin influx and then activates conjugate hydrolyase enzymes to release active IAA; however, this is insufficient to keep the AZ insensitive to ethylene and prevent abscission, which usually only lags the initial abscission by several hours. For complete inhibition of abscission, exogenous auxin is required. In this study, abundant IAA-Ala and IAA-Ile could completely inhibit abscission, while IAA-Asp had little effect. As previously reported, IAA-Ala possesses auxin activity without hydrolysis, so we chose to use IAA-Ile to study the effects of an auxin conjugate on abscission. IAA–Asp is thought to be involved in a degradation pathway and, because it does not release free auxin, it has a minimal role in abscission. In auxin-depleted induced tomato pedicel abscission, IAA in the AZ declined during the early stages (before 8 h) when abscission was initialized, then increased at 16 h, which is an ethylene-accelerated abscission stage. About 60% percent of the flowers abscised during this stage. The low auxin concentration in the AZ is linked to increased ethylene sensitivity, and might also result in secondary cell wall deposition at the site of the reduced auxin response, which is instrumental during the separation process [38, 39]. The different times of treatment showed that before 6 h, IAA-Ile could effectively delay abscission, while there was little effect after 8 h. Moreover, incubation with IAA-Ile for 2 h was sufficient to inhibit abscission. These results showed that there is not enough auxin conjugate in the AZ to inhibit abscission, and that the optimal time to inhibit abscission by exogenous auxin conjugate application is before 6 h.

To determine whether de novo synthesized auxin conjugates are required for abscission, the tomato pedicel explants were incubated in MS agar for different times then transferred to IAA-Ile medium containing 10 mM CHX (cycloheximide). Inhibition of de novo synthesis of auxin conjugate hydrolase enzymes would decrease the auxin concentration and accelerate abscission. Our results showed that induced auxin conjugate hydrolases are required for the release of more free auxin to delay abscission. The results also indicated that there is not enough auxin conjugate hydrolase activity before 4 h and insufficient levels of auxin conjugate after 8 h. In our previously study, the early abscission stage was found before 8 h, and the dynamics of free auxin in the AZ seems to be a major regulator for this process [22].

### Expression of tomato *SlILL* genes during abscission

Assaying *SlILL* gene expression in the different tomato organs showed that *SlILL1*, *3*, *4*, and *7* are expressed at a relatively low levels, while the expression of *SlILL2* and *5* is somewhat higher in the AZ compared with that in other organs. Notably, only SlILL6 had a high level of expression in the AZ. Moreover, *SlILL1*, *5*, and *6* showed unique expression patterns in the AZ, and the expression of all three genes was altered by 1-MCP and auxin treatments, showing that gene expression is highly associated with abscission. A previous report showed that wounding could induce the expression of auxin conjugate hydrolases, such as *IAR3* in *Arabidopsis*, and a nonspecific increase in expression of both *SlILL*3 and 7 in the AZ and NAZ might be a manifestation of the wound response. However, in another view, *SlILL1*, *3*, *5*, *6*, and *7* all showed a trend of increasing expression during abscission in the AZ, which may not exclude their potential role in releasing auxin to delay abscission. This also draws attention to the fact that expression of all *SlILL* genes is inhibited under auxin treatment. The expression of *SlILL* genes is mediated by free auxin, which might be required for precise auxin signaling in mediating AZ abscission. In addition, the auxin conjugate hydrolase enzymes are regulated by the free auxin content at the transcriptional level, which might be important to determine the precise auxin concentration required for cell development and for responses to environmental stimuli.

### Transcription and translation of tomato SlILLs protein during abscission

*SlILL1*, *5*, and *6* showed special expression profiles during abscission; therefore, they were selected as abscission-related auxin conjugate hydrolase genes for western blotting analysis. The pattern of SlILL1, SlILL5 and SlILL6 protein levels in response to 1-MCP and IAA indicated that they were possibly related to abscission.

### *SlILL1, 5*, and *6* are major regulators of delayed abscission

The plants in which single *SlILL* genes were silenced by VIGS showed a normal phenotype, but had reduced sensitivity to specific auxin conjugates. The more hydrolase genes that were downregulated, the less sensitive the plants became to exogenous auxin conjugates [11, 40]. Double and triple mutants of Arabidopsis auxin conjugate hydrolases showed lower auxin levels in seedlings and defective responses to exogenous auxin conjugates [11, 26]. In this study, all tomato hydrolases showed distinct expression patterns and cleavage activities, implying that they might have distinct, yet overlapping, functions during abscission. Silencing *SlILL1,* SlILL5, and *SlILL6* individually resulted in significantly accelerated abscission in pedicel explants, while double silencing showed that *SlILL3* and *7* had little effect on abscission. The double-silenced *SlILL1 + 5*, *SlILL1 + 6*, and *SlILL5 + 6* explants showed a significant increase in abscission compared with that of the single gene-silenced explants, implying that *SlILL1*, *5*, and *6* play important roles in the abscission process. The triple-silenced lines further supported this hypothesis, since they showed a further accelerated abscission rate compared to the double-silenced VIGS lines. Taken together, these results indicated that the auxin conjugate hydrolase enzymes encoded by *SlILL1, SlILL5*, and *SlILL6* coordinate to mediate pedicel abscission in tomato. We also used VIGS*1 + 5 + 6* lines to investigate restoring the phenotype, The result showed that IAA addition could restore the phenotype of the VIGS lines (Additional file 5: Figure S3).

The concept that the AZ local auxin concentration plays a negative role in mediating abscission procession has been understood for 50 years. Artificial activation of the bacterial auxin biosynthetic genes iaaL and iaaM in the AZ further supports this view [41]. Moreover, auxin is transported in a polar manner to other organs mediated by PIN efflux carriers that determine the direction of flux [42]. It is also important to prevent abscission by maintaining this constant auxin flux [43]. The effect of auxin on PIN expression has been well- studied. Below the optimal concentration, the auxin-induced PIN expression effect was correlates positively with the amount of auxin, while PIN expression was reduced when the auxin concentration was far in excess of the optimal concentration. In AZ, the auxin concentration is relatively low, and depressed SlILR1, 5, and 6 expression in the triple silenced line showed further downregulation of the AZ auxin concentration, leading to a low expression of PIN3, 4, and 8, which might decrease auxin polar transport [44]. The mechanism by which SlILL1, SlILL5, and SlILL6 mediate abscission, not only via altered auxin content, also via auxin flux by affecting PIN gene expression. It is reasonable to propose that the local auxin content in the AZ and auxin transport are tightly connected to regulate abscission.

## Conclusions

In this study, we identified ILR genes in the tomato genome. Their protein structures and activities were also characterized, their expression level and protein accumulation in different abscission stage were also investigated using qRT-PCR and western blotting, respectively. Further, three candidates ILR genes were identified to function in tomato abscission. Our identification and characterization of ILRs genes in tomato revealed that certain ILRs might fine-tune the levels of auxin in tomato AZ, which is critical for abscission. These findings provide a new way to inhibit abscission by applying auxin conjugations to flowers.

## Methods

### Plant material

The *Solanum lycopersicum* cultivar ‘Zhongshu NO 6’ and transgenic line ‘DR5’ were grown in soil in a greenhouse (25 ± 3 °C day, 15 ± 3 °C night) under natural light. Newly opened flowers with fresh yellow petals at an opening angle of 90° were screened to obtain pedicel explants. The explants were trimmed to 2 cm in length and contained the proximal side, the abscission zone, and the distal side of the flower after the petals and base were removed.

### Auxin, 1-MCP, and auxin-conjugate treatments, and abscission rate assessment

Fifty explants were inserted by their proximal ends into 1% w/v agar medium in a 40 × 25 × 20 cm glass container at 25 °C. The agar medium contained deionized water (control) or 50 μg·g^− 1^ IAA in IAA treatment (dissolved in ethanol and the control also contain a equal amount of ethanol), and for phenotype restoration experiment in the VIGS lines, the IAA were added at 10 μg·g^− 1^ and 50 μg·g^− 1^ respectively. For the 1-MCP treatment, 1-MCP (20 μl l^− 1^) was applied for 24 h to treat the whole plant before the flowers were removed and incubated. For auxin conjugate treatments, the agar medium was supplemented individually with 10 to 100 μM IAA-Ala, IAA-Asp, and IAA-Ile (dissolved in ethanol and the control also contain equal amounts of ethanol). The explants were incubated in medium containing IAA-Ile for 1, 2, 4, 6, 8, and 12 h then transferred to the 1% w/v agar medium without IAA conjugate to investigate abscission. The explants were also incubated in control medium (without IAA conjugate) for 1, 2, 4, 6, 8, and 12 h then transferred to medium containing the IAA conjugate. To determine whether the IAA conjugate hydrolytic enzymes depended on de novo synthesis during abscission, the explants were first incubated in medium agar for 1, 2 and 4 h and then transferred to IAA conjugate medium containing 5 μm cycloheximide (CHX). Pedicel abscission rates were recorded every 8 h for 40 h, as described by Wang et al. (2005).

### Identification of auxin conjugate hydrolase genes

To find previously identified *ILL* family genes in tomato, “auxin conjugate hydrolase” was used as a query to search the SGN database (http://solgenomics.net). We also used the amino acid sequences of the conserved ILL domains from known ILL proteins (including AtILLs (*Arabidopsis*), MeILLs (*Medicago truncatula)* and BrILLs (*Brassica rapa*)) as queries to identify all members of the tomato ILL family. DNASTAR and FGENESH (http://www.softberry.com/) were then used to analyze and predict the unknown SlILLs. Putative open reading frames and functional domains were identified using ORF finder and BLASTP searches (blast.ncbi.nlm.nih.gov), respectively.

### Isolation of full-length ILL cDNA sequences using RT-PCR

Total RNA was extracted from tomato seedlings using the TRIZOL reagent (Invitrogen, Carlsbad, CA, USA). First-strand cDNA was synthesized using a reverse transcription system. The seven novel full-length *SlILL* cDNA sequences were amplified by PCR using specific primers (Additional file 6: Table S1). The PCR conditions consisted of denaturation at 94 °C for 4 min; followed by 30 cycles of 30 s at 94 °C, 30 s at 55 °C, and 120 s at 72 °C; with a final 10-min elongation at 72 °C.

### Multiple sequence alignments and phylogenetic analysis

DNAStar software and the web service ExPASy Proteomics Server (http://ca. expasy.org) were used to analyze the gene sequences. Multiple sequence alignments were performed using Clustal X v1.81 [45]. Phylogenetic analysis was performed using the neighbor-joining (NJ) method as implemented in the MEGA 4.1 program [46]. Conserved motifs were identified using Clustal W.

### RNA extraction and expression analysis

Total RNA was extracted from AZ and NAZ (the proximal part) using the TRIZOL reagent (Invitrogen). Quantitative real-time reverse transcription PCR (qRT-PCR) assays were performed as described by Jain [47]. First-strand cDNA was synthesized using the High Capacity cDNA Archive Kit (Applied Biosystems, Foster City, CA, USA) in reactions containing 2 μg of total RNA. For qRT-PCR, each 20 μl reaction system contained 1 μl of cDNA, 200 nM of each primer, and SYBR Green PCR Master Mix (Qiagen) (Additional file 4: Table S1). Amplifications were performed on an ABI 7500 fast system (Applied Biosystems). Two independent RNA samples and three technical replicates for qRT-PCR were performed for each sample.

### Protein extraction and Western blotting analysis

For western blotting, synthetic peptides: FFFLCIFLLVLTS, SSYFDQEFVKQIL, and DCSIWTKECSNEI were used to raise polyclonal antibodies targeting SlILL1, SlILL5, and SlILL6, respectively, by Abmart (Berkeley Heights, NJ, USA). The ß-actin conjugated antibody was purchased from Abbkine Company (Wuhan, China) and the anti-rabbit conjugated antibody was purchased from Sigma (St. Louis, MI, USA. Total proteins were extracted from the AZ (control and 1-MCP treatment) at 0, 2, 4, and 16 h using the Minute™ total protein extraction kit (Invent Biotechnologies, Plymouth, MN, USA). The protein concentration was assessed using the BCA assay (Takara company). Each sample was boiled for 5 min with loading buffer, separated in a 10% SDS-PAGE, and transferred to polyvinylidene difluoride (PVDF) membranes (Bio-Rad, Hercules, CA, USA). Then, the PVDF membranes were blocked with 5% non-fat dried milk for 2 h at room temperature, probed with the primary antibodies at 4 °C overnight, and then with anti-rabbit antibody for 1 h at room temperature. Chemiluminescent detection was performed on an Azure c600 system (Azure Biosystems, Dublin, CA, USA) (Additional file 7). Quantitative analysis of the western blotting data was performed using Image J software of selected gel band intensities.

### Synthesis and purification of his-tagged fusion proteins

The amplified cDNAs were cloned into the pET 30a (+) expression vector, which contains both N-terminal and C-terminal His tags for heterologous expression in *Rosetta Escherichia coli*. Single colonies of transformed and untransformed *E. coli* TOP10 (as control), were inoculated into 150 ml of Luria-Bertani broth containing 50 μg·ml^− 1^ ampicillin and grown at 28 °C overnight.

Isopropyl β-D-1-galactopyranoside was used at a concentration of 1 mM to induce the *E. coli* cultures, which were incubated at 28 °C for 8 h. The cells were recovered by centrifugation and resuspended in 1.5 ml lysis buffer (50 mM NaH_2_PO_4_, 300 mM NaCl, 10 mM imidazole, 50 μg·ml^− 1^ phenylmethanesulfonyl fluoride, 1 mg·ml^− 1^ lysozyme, 0.1% Triton; pH 8.0) For enrichment of the tagged proteins, samples were incubated on ice for 30 min, then frozen and thawed three times in liquid nitrogen. *E. coli* DNA was removed by adding 500 U DNAse I (Takara) and incubating at room temperature for 20 min. After centrifugation, the cell lysate was transferred to a new tube, and 20 μl Ni-NTA Agarose (Qiagen, Valencia, CA, USA) was added. The lysate was then incubated with rotation for 1 h at 4 °C and the agarose was subsequently washed three times with wash buffer (300 mM NaCl, 50 mM NaH_2_PO_4_ and 20 mM imidazole; pH 8.0). Proteins were eluted three times with 30 μl of elution buffer (300 mM NaCl, 50 mM NaH_2_PO_4_, 250 mM imidazole; pH 8.0). The proteins were analyzed using denaturing polyacrylamide gel electrophoresis (SDS–PAGE) on 12% gels and quantified using the BCA assay Protein Quantification Kit (Beyotime, Jiangsu, China).

### IAA conjugate hydrolase enzyme assays

For the enzyme assays, 2 μg samples of the enriched protein fraction were mixed with assay buffer (100 mM Tris, 200 μM MnCl_2_, 1 mM DTT; pH 8.0), and the IAA conjugates, IAA-Ala, IAA-Leu, IAA-Asp, IAA-Gly and IAA-Trp, were added individually at a concentration of 1 mM, as substrates. The reactions were incubated at 37 °C for 1 h and terminated by the addition of 20 μl 1 N HCl. The aqueous phases were then extracted with 250 μl of ethyl acetate, evaporated, and the reaction products were dissolved in 100 μl of methanol for ultra performance liquid chromatography (UPLC) analysis. Aliquots (0.2 μl) of the methanol extracts were then injected into a UPLC instrument (Waters H-class, Waters, Millford, MA, USA) coupled to an autosampler (Jasco AS-1550), and equipped with a 2.1 × 100-mm Cortecs C18 2.7 μm, reversed phase column. 1% aqueous acetic acid and 100% methanol were used as the solvents. The solvent program was 15% methanol for 3 min, followed by a gradient from 15 to 90% methanol for 4 min, then a decreasing gradient from 90 to 15% for 3 min with 1% acetic acid throughout. Compounds were detected using ACQUITY FLR fluorescence (excitation 278 nm, emission 350 nm) and quantitated by the peak area.

### Beta glucuronidase (GUS) staining

GUS assay was performed by incubating tissues from DR5::GUS plants in 50 mM sodium phosphate buffer (pH 7.0) containing 0.4 mg 5-bromo-4-chloro-3-indolyl-b-D-glucuronicacid (X-Gluc), 1 mM potassium ferricyanide, 1 mM potassium ferrocyanide and 0.5% Triton X-100 for 12 h at 37 °C. The samples were then incubated in 70% ethanol to remove chlorophyll. Photographs were obtained using a digital camera under a Nikon Eclipse 80i microscope.

### Auxin concentration measurements

The IAA concentration was measured using gas chromatography-mass spectrometry (GC-MS) according to the method described by Barkawi. In short, 100 μl of extraction buffer (65% isopropanol, 35% 0.2 M imidazole, pH 7.0) that contained 5 ng of a [^13^C_6_] (Isoreag, Shanghai, China) IAA standard from 100 mg of homogenized frozen tissue. Tissue were centrifuged at 14,000×*g* for 5 min after incubation on ice for 1 h, using solid phase extraction and ethereal diazomethane for the methylated forms. GC-MS (Thermo, USA) was run in select ion monitoring mode and IAA was quantified using isotope dilution analysis [48].

### Virus-induced gene silencing of the SlILL genes

The 300-bp DNA fragments derived from each of the seven *SlILL* genes were synthesized by the Shenggong Company (Shanghai, China). The individual fragments encompass nucleotides 303–602 for *ILL1*, 1–300 for *ILL2*, 343–642 for *ILL3*, 230–529 for *ILL4*, 18–317 for *ILL5*, 250–549 for *ILL6*, and 1–300 for *ILL7*. The synthetic fragments were cloned into the tobacco rattle virus (TRV) vector pTRV2, resulting in pTRV2-*ILL1*, −*ILL3*, −*ILL5*, −*ILL6*, and -*ILL7* constructs. In addition, the fragment from *ILL1* was subcloned into the pTRV2-*ILL5* construct to generate pTRV2-*ILL1* + *ILL5*, the fragments from the *ILL1* and *ILL5* genes were subcloned into pTRV2-*ILL6* to generate the pTRV2-*ILL1* + *ILL6*, pTRV2-*ILL5* + *ILL6*, and pTRV2-*ILL1* + *ILL5* + *ILL*6 constructs, and the *ILL3* gene fragment was subcloned into pTRV2-*ILL7* to generate the pTRV2-*ILL3* + *ILL7* construct. Tomato plants were infected with a mixed culture of *Agrobacterium tumefaciens* containing the pTRV1 vector and the above recombinant TRV2 vectors. The VIGS infection method has been described in detail previously (Jiang et al. 2008 [49]).

## Additional files

Additional file 1: **Figure S1** Sequence alignment of predicted tomato SlILL proteins obtained using the ClustalX program. Amino acid residues identical in at least four of the six sequences are shown in gray, and highly conserved residues are shown in black. Gaps in the sequences, introduced to maintain alignment, are indicated by dots. The peptidase M20 domain is shown by a solid dark line, and the dotted line indicates the M20 dimerization domain. (TIF 504 kb)

Additional file 2: **Figure S2** Denaturing gel electrophoresis of the induced and enriched His-tagged SlILL proteins used in the enzyme activity assays. Molecular mass standards are shown in the flanking lanes. (TIF 1089 kb)

Additional file 3: Analytical parameters of the compound IAA: correlation coefficient (R), linear range, calibration curves and limits of detection and quantification (LOD, LOQ) of IAA. (DOCX 12 kb)

Additional file 4: The raw data of GC-MS IAA content. (XLSX 10 kb)

Additional file 5: Effects of auxin on the abscission rate of VIGS1+5+6 pedicel explants: (filled diamond) wild-type (WT); (filled square) VIGS; (filled triangle) WT incubated in 10 μg•g-1 IAA agar; (filled circle) VIGS incubated in 10 μg•g-1 IAA agar; (empty triangle) WT incubated in 50μg•g-1 IAA agar; (empty circle) VIGS incubated in 50 μg•g-1 IAA agar. The results are means of three replicates (60 flowers each) ± SE. The agar medium contained deionized water (control) or was supplemented with 10 μg•g-1 IAA or 50 μg•g-1 IAA. (TIF 1236 kb)

Additional file 6: Primer for qRT-PCR and fusion protein. (DOC 29 kb)

Additional file 7: The raw data of western blot chemiluminescent detection. (TIF 408 kb)

## Data Availability

The datasets supporting the results of this publication are included within the article and Additional files 1, 2, 3, 4, 5, 6, 7.

## Abbreviations

1-MCP: 1-methylcylopropene
AR: Abscission rates
AZ: Abscission zone
BLAST: Basic local alignment search tool
cDNA: Complementary DNA
CHX: Cycloheximide
ELISA: Enzyme-linked immunosorbent assay
ER: Endoplasmic reticulum
GUS: Beta-glucuronidase
IAA: Indole-3-acetic acid
IBA: Indole-3-butyric acid
ORFs: Open reading frames
PVDF: Polyvinylidene difluoride
qRT-PCR: Quantitative real-time reverse transcription polymerase chain reaction
SDS-PAGE: Sodium dodecyl sulfate polyacrylamide gel electrophoresis
SGN: Solgenomics
UPLC: Ultra Performance Liquid Chromatography
VIGS: Virus-induced gene silencing
