# Supplementary figures and images for: A family of auxin conjugate hydrolases from Solanum lycopersicum and analysis of their roles in flower pedicel abscission
Source: BMC Plant Biol. 2019 Jun 3;19:233. doi: 10.1186/s12870-019-1840-9 (PMC6547480; doi:10.1186/s12870-019-1840-9)

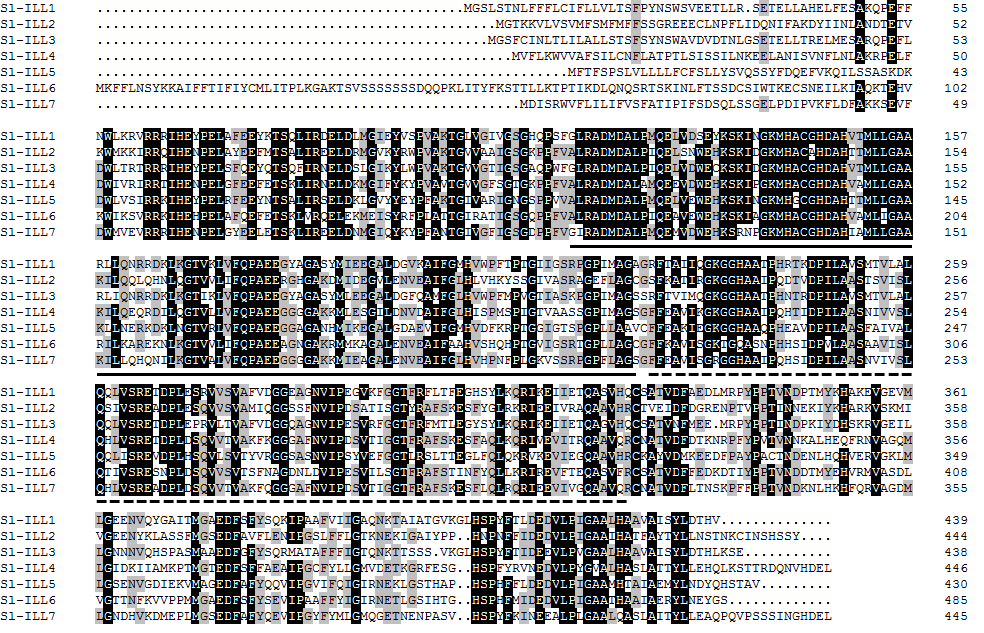

Supplement: Supplementary file 1 — Figure S1 Sequence alignment of predicted tomato SlILL proteins obtained using the ClustalX program. Amino acid residues identical in at least four of the six sequences are shown in gray, and highly conserved residues are shown in black. Gaps in the sequences, introduced to maintain alignment, are indicated by dots. The peptidase M20 domain is shown by a solid dark line, and the dotted line indicates the M20 dimerization domain. (TIF 504 kb) [file 12870_2019_1840_MOESM1_ESM.tif]

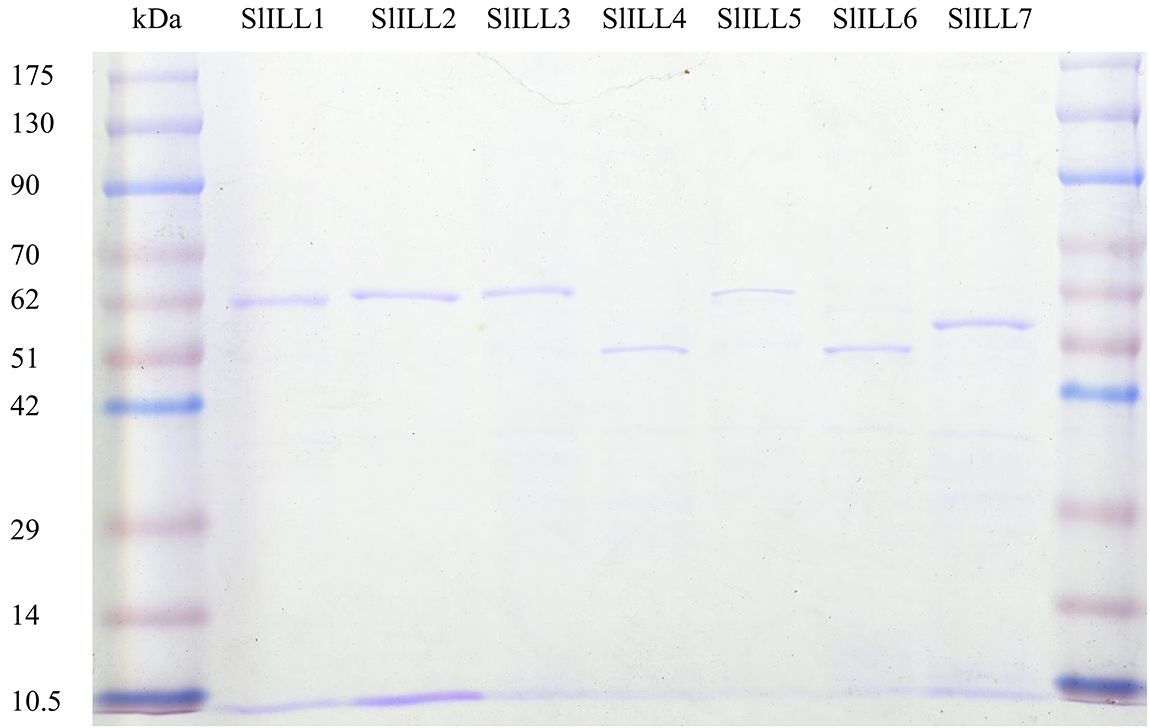

Supplement: Supplementary file 2 — Figure S2 Denaturing gel electrophoresis of the induced and enriched His-tagged SlILL proteins used in the enzyme activity assays. Molecular mass standards are shown in the flanking lanes. (TIF 1089 kb) [file 12870_2019_1840_MOESM2_ESM.tif]

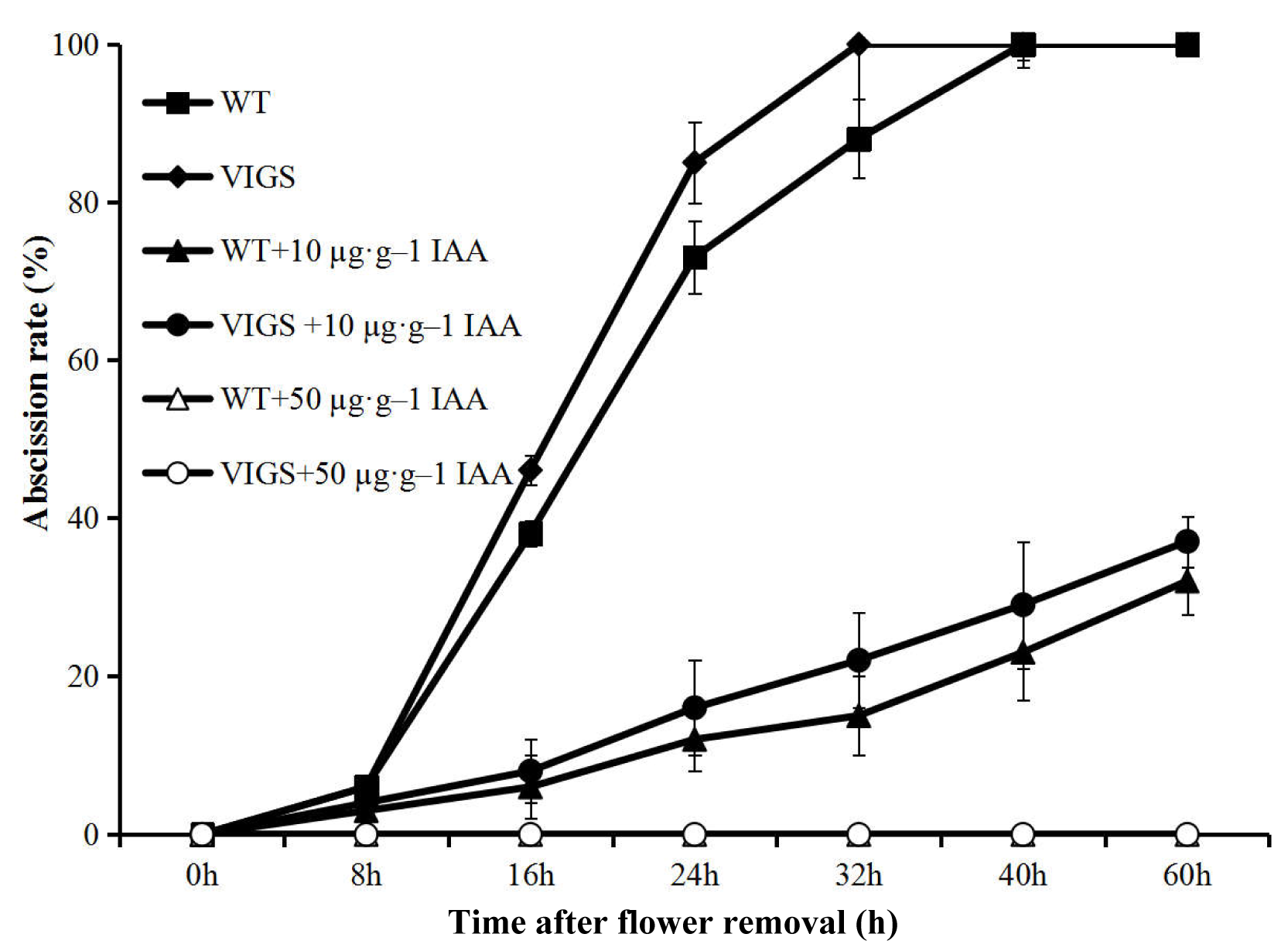

Supplement: Supplementary file 5 — Effects of auxin on the abscission rate of VIGS1+5+6 pedicel explants: (filled diamond) wild-type (WT); (filled square) VIGS; (filled triangle) WT incubated in 10 μg•g-1 IAA agar; (filled circle) VIGS incubated in 10 μg•g-1 IAA agar; (empty triangle) WT incubated in 50μg•g-1 IAA agar; (empty circle) VIGS incubated in 50 μg•g-1 IAA agar. The results are means of three replicates (60 flowers each) ± SE. The agar medium contained deionized water (control) or was supplemented with 10 μg•g-1 IAA or 50 μg•g-1 IAA. (TIF 1236 kb) [file 12870_2019_1840_MOESM5_ESM.tif]

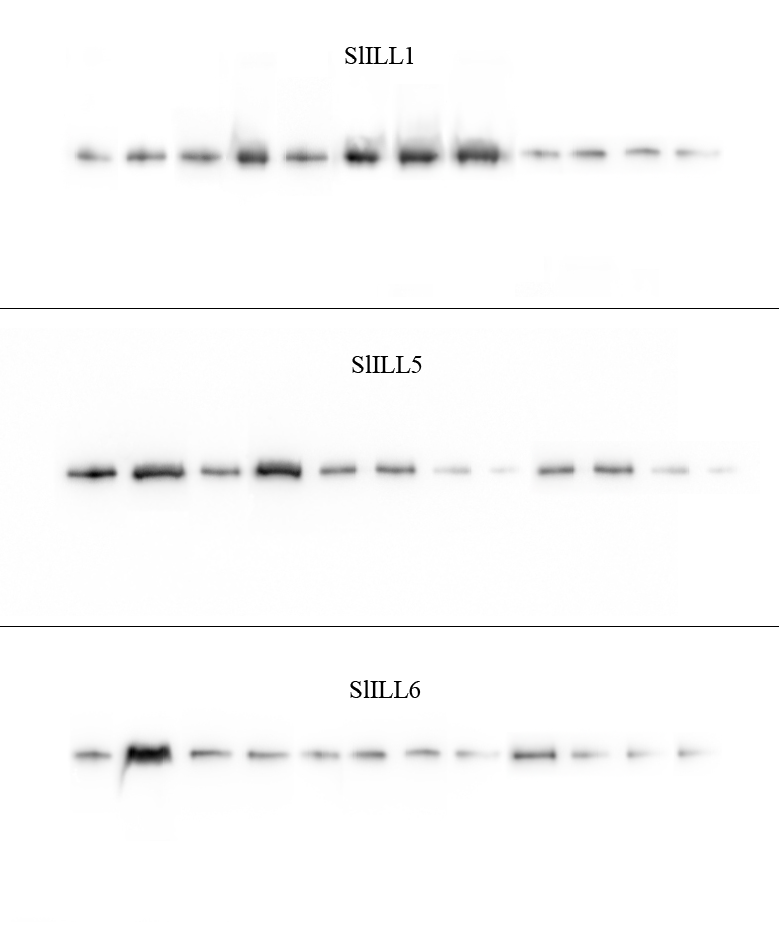

Supplement: Supplementary file 7 — The raw data of western blot chemiluminescent detection. (TIF 408 kb) [file 12870_2019_1840_MOESM7_ESM.tif]
