# Supplementary material for: A family of auxin conjugate hydrolases from Solanum lycopersicum and analysis of their roles in flower pedicel abscission
Source: BMC Plant Biol. 2019 Jun 3;19:233. doi: 10.1186/s12870-019-1840-9 (PMC6547480; doi:10.1186/s12870-019-1840-9)
Supplement: Supplementary file 3 — Analytical parameters of the compound IAA: correlation coefficient (R), linear range, calibration curves and limits of detection and quantification (LOD, LOQ) of IAA. (DOCX 12 kb) [file 12870_2019_1840_MOESM3_ESM.docx]

**Table 1**

Analytical parameters of the compound IAA: correlation coefficient (R), linear range, calibration curves and limits of detection and quantification (LOD, LOQ) of IAA.

| Compound | Calibration curves | Linear range (ng/mL) | R^2^ | LOD (ng/mL) | LOQ (ng/mL) |
| --- | --- | --- | --- | --- | --- |
| IAA | y=3.06507*10^-5^x + 0.0892068 | 312.5-5000 | 0.99 | > 312.5 | > 312.5 |
