# Supplementary material for: A family of auxin conjugate hydrolases from Solanum lycopersicum and analysis of their roles in flower pedicel abscission
Source: BMC Plant Biol. 2019 Jun 3;19:233. doi: 10.1186/s12870-019-1840-9 (PMC6547480; doi:10.1186/s12870-019-1840-9)
Supplement: Supplementary file 6 — Primer for qRT-PCR and fusion protein. (DOC 29 kb) [file 12870_2019_1840_MOESM6_ESM.doc]

**Primer for qRT-PCR**

*SLILL*1-F, 5’-TAGATGGTGGTGAGGCTGGA-3’;

*SLILL*1-R, 5’-CGCCATACTGCACGTTTTCC-3’;

*SLILL*2-F, 5’-CTAAAACCGGTGTCGTTGCC-3’;

*SLILL*2-R, 5’-ATATCTTTAGCCCCGTGCCC-3’;

*SLILL*3-F, 5’-TCTGGCCATTTATGCCCGTT-3’;

*SLILL*3-R, 5’-ACATTACCGGCTTGACCACC-3’;

*SLILL*4-F, 5’-ACACGATGCCCATGTTGCTA-3’;

*SLILL*4-R, 5’-AAAGAAGCCACTTCCAGCCA-3;

*SLILL*5-F 5’-GCATTGCAGCAGCTCATCTC-3’;

*SLILL*5-R, 5’-ACGTTCTCCGAACCAAGCAT-3’;

*SLILL*6-F 5’-TGCTGGCTGTGGCTTCTTTA-3;

*SLILL*6-R, 5’-CGTTCACAGTCGGAGGGTAG-3;

*SLILL*7-F 5’-GGTCTGCATGTCCATCCCAA-3;

*SLILL*7-R, 5’-TCGTTGCCTAAGCTGCAGAA-3;

**Primer for fusion protein**

*SLILL*1 F:5’-CCC*GAATTC*GTAGAAGAGACCCTTTTGAGATCTGAAAC-’3

*SLILL*1 R:5’-CCC*GTCGAC*GGATGATTCTTCAATCTCCAAAAATGTAT-’3

*SLILL*2 F:5’- CCC*GAATTC*CGCGAAGAAGAATGTTTGAATCCAT-’3

*SLILL*2 R:5’-GG*GTCGAC*GGACTAGACCTAATGATCATCTTCTTGCAT-’3

*SLILL*3 F:5’-GG*GAATTC*GTAGATGTAGATACAAATTTGGGATCTGAAA-’3

*SLILL*3 R:5’-CC*GTCGAC*TCTGCTAATAAATAGAGTCAAATCTCAACGT-’3

*SLILL*4 F:5’-GG*GAATTC*ATTTCTTCTATTTTGAACAAAGAGGAGTTG-’3

*SLILL*4 R:5’-GG*GTCGAC*ATTATTTTCATCCGCAAGGTCTCTCA-’3

*SLILL*5 F:5’-CCC*GAATTC*AGCTACTTTGATCAAGAATTTGTTAAGCA-’3

*SLILL*5 R:5’-CCC*GTCGAC*TGGTCATCACCGCTTTAGATTTGCTTATT-’3

*SLILL*6 F:5’-CCC*GAATTC*ATGAAGTTTTTTCTAAATTCTTATAAAAAAGCAATC-’3

*SLILL*6 R:5’-CCC*GTCGAC*CGATAAGTGAGGCCTGCTCAAGTAGTT-’3

*SLILL*7 F:5’-CCC*GAATTC*CAATTGAGTTCAGGAGAACTACCAGACAT-’3

*SLILL*7 R:5’-CCC*GTCGAC*CAAAAGAGTAGCACCTCACAATTCATCGT-’3
